# The Serpentine Illusion: A Visual Motion Illusion Induced by Phase-Shifted Line Gratings

**DOI:** 10.3389/fnins.2020.612153

**Published:** 2020-12-07

**Authors:** Junxiang Luo, Zheyuan Chen, Yiliang Lu, Lothar Spillmann, Ian Max Andolina, Wei Wang

**Affiliations:** ^1^Institute of Neuroscience, State Key Laboratory of Neuroscience, Key Laboratory of Primate Neurobiology, CAS Center for Excellence in Brain Science and Intelligence Technology, Chinese Academy of Sciences, Shanghai, China; ^2^Shanghai Center for Brain Science and Brain-Inspired Intelligence Technology, Shanghai, China; ^3^Department of Neurology, University of Freiburg, Freiburg im Breisgau, Germany; ^4^University of Chinese Academy of Sciences, Beijing, China

**Keywords:** motion illusions, perception, energy model, equiluminance, motion perception, illusion

## Abstract

In a pattern of horizontal lines containing ± 45° zigzagging phase-shifted strips, vivid illusory motion is perceived when the pattern is translated up or down at a moderate speed. Two forms of illusory motion are seen: [i] a motion “racing” along the diagonal interface between the strips and [ii] lateral (sideways) motion of the strip sections. We found the relative salience of these two illusory motions to be strongly influenced by the vertical spacing and length of the line gratings, and the period length of the zigzag strips. Both illusory motions are abolished when the abutting strips are interleaved, separated by a gap or when a real line is superimposed at the interface. Illusory motion is also severely weakened when equiluminant colored grating lines are used. Illusory motion perception is fully restored at < 20% luminance contrast. Using adaptation, we find that line-ends alone are insufficient for illusory motion perception, and that both physical carrier motion and line orientation are required. We finally test a classical spatiotemporal energy model of V1 cells that exhibit direction tuning changes that are consistent with the direction of illusory motion. Taking this data together, we constructed a new visual illusion and surmise its origin to interactions of spatial and temporal energy of the lines and line-ends preferentially driving the magnocellular pathway.

## Introduction

The Italian psychologist Gaetano Kanizsa described a thin illusory contour running at right angles between two juxtaposed, phase-shifted line gratings, similar to a real line but with no physical correlate in the inducing pattern (Kanizsa, 1974). The illusory contour separating the two gratings could be straight or curved depending on the shape of the grating pattern that produced it (Kanizsa, 1976, 1979). Historically, patterns eliciting illusory contours have been well studied psychophysically (Spillmann and Dresp, 1995; Soriano et al., 1996; Halko et al., 2008). Physiologically, Von der Heydt et al. (1984) and Von der Heydt and Peterhans (1989) studied the neural response of V2 units in the monkey to abutting line gratings. They found neurons that responded to these patterns similarly to real lines (i.e., with consistent orientation selectivity). Subsequent neurophysiological, imaging, and computational modeling studies further extended the knowledge of the neuronal mechanisms that mediate the perception of illusory contours (Ramsden et al., 2001; Montaser-Kouhsari et al., 2007; Schmid, 2008; Murray and Herrmann, 2013; Cohen et al., 2014).

**FIGURE 1:**
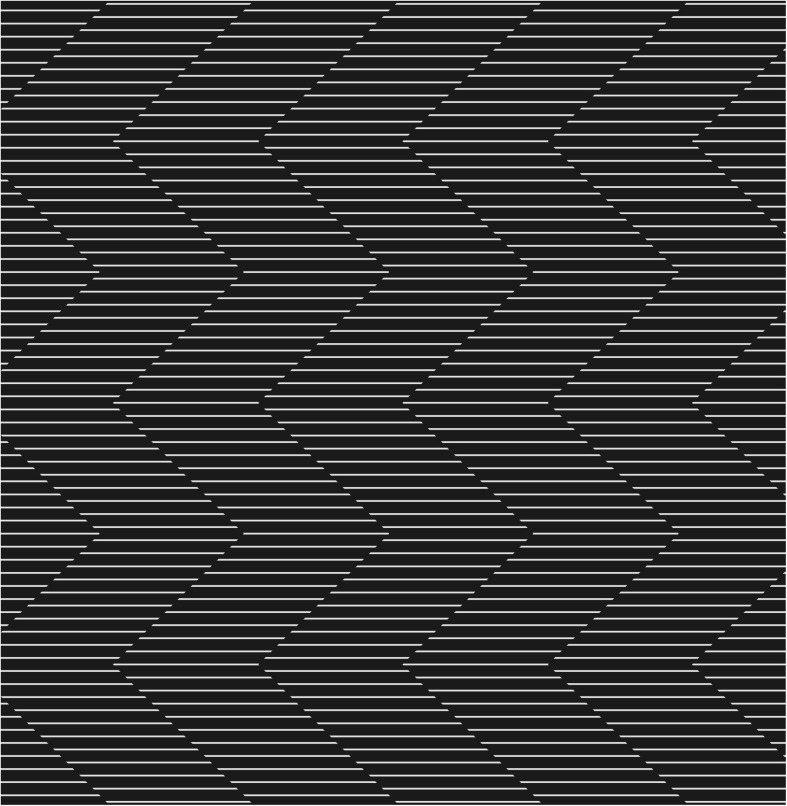
The Serpentine Illusion. Two kinds of illusory motion can be seen when this pattern is moved up and down (you can fixate on a pen or finger moving up and down over the figure): (i) diagonal motion along the zigzag interface between the phase-shifted grating strips; and (ii) lateral (sideward) motion parallel to the line gratings.

Previous psychophysical studies used gratings that abut each other at right angles to the illusory contour. By comparison, we used a pattern where the interface between two grating strips is alternated at ± 45° relative to the horizontal line gratings (Figure 1 and Supplementary Video 1). When this pattern is moved up and down, vivid illusory motion can be seen both along the illusory contours and parallel (sideward) to the horizontal line gratings. The motion along the illusory contour may best be described as “racing” along the diagonal paths between the phase-shifted grating strips. The sideward motion resembles a horizontal or lateral shift to one or the other side of the grating strips. We refer to these two kinds of illusory motion as “diagonal” and “lateral,” respectively. The illusory motions are reminiscent of the movement of a snake, where lateral motion of the body segments combines with diagonal forward motion. We therefore name this effect the “Serpentine Illusion.”

Both kinds of illusory motion can be perceived simultaneously, although at different strength, depending on the parameters defining the abutting gratings. In this study, we wish to quantify the stimulus parameters that influence both kinds of illusory motion. We first investigate how the direction of stimulus motion (upward or downward), and configurational modifications such as separating and interleaving the grating strips, affect the Serpentine Illusion (Experiment 1). Next, we identify the parametric values such as the length of line gratings, vertical spacing between the grating lines, and vertical length of one zigzag period, that produce optimal diagonal, diagonal and lateral, and lateral illusory motion (Experiment 2). We attempt to delineate the neuronal mechanisms underlying these illusions by testing at equiluminance (Experiment 3), varying the figure-ground contrast (Experiment 4) and adapting to selected stimulus features (Experiment 5). Finally we test the illusory stimuli using a spatiotemporal energy model of V1 neurons (Adelson and Bergen, 1985; Baker and Issa, 2005; An et al., 2012), to quantify if their direction tuning is affected by Serpentine motion in a way consistent with the illusory motion perception observed by human subjects.

## Materials and Methods

### Subjects

A total of nine subjects, 6 males and 3 females, aged from 20 to 30 years, participated as observers. Not all of them served in each experiment. All subjects were naïve to the experiments at the start of the experiment and had normal or corrected-to-normal vision. Experiments were approved by the Ethics Committee of the Institute of Neuroscience, Chinese Academy of Sciences. Subjects gave their consent to the procedure and institutional guidelines. This work adheres to the tenets of the Declaration of Helsinki.

### Experimental Set-Up and Testing Procedure

Stimulus patterns were displayed on the screen of a SONY CPD-G520 CRT monitor 57 cm away from the subjects’ eyes, corresponding to a visual angle of 30° × 40°. The refresh rate of the monitor was 100 Hz. All stimuli were generated using the Psychophysics toolbox (Kleiner et al., 2007) running under MATLAB. The gamma value of the monitor was corrected so that the luminance was linearly distributed across the screen for all gray shades using a ColorCal colorimeter (Cambridge Research Systems, Cambridge, United Kingdom). Subjects sat in a dark room with their head stabilized by a head- and chin-rest. They maintained fixation binocularly on a central red point, 0.3° in diameter for the duration of every experiment performed. Subjects could repeat each trial until they were confident of their choice. Thereafter they were asked to describe the perceived illusory motion or match the perceived motion(s) to a choice panel on the screen by pressing a keyboard button (please see the experimental procedure in each experimental section for a detailed description). This study was divided into 5 experiments.

## Results

### Experiment 1. Modes of Illusory Motion

#### Stimuli

The stimulus pattern was derived from the standard pattern shown in Figure 1. It consisted of grating strips of white lines on a black background, phase-shifted by half a cycle and abutting each other along a diagonal, zigzagging interface. The white lines had a luminance of 96 cd/m^2^ and the black background a luminance of 0.15 cd/m^2^. Individual grating lines were spaced 0.67° apart vertically, were 2.5° long, and were progressively shifted 45° sideward in one or the other direction to form zigzags with a period length of 5°. The pattern was moved up or down on the monitor at a speed of 5°/s while subjects maintained fixation. To test the effects of the local interface of phase-shifted lines, we created variants that interleaved or separated the line-ends by 0.3°, and we also created a pattern with physical lines of the same width superimposed over the position of the illusory contours. There was no time limit for observation and subjects were free to continue observing until they were confident of their perceptual report.

#### Procedure

Six naïve subjects participated in the test. They were asked to verbally describe if they saw any motion in addition to the physical upward/downward motion.

#### Results

**FIGURE 2:**
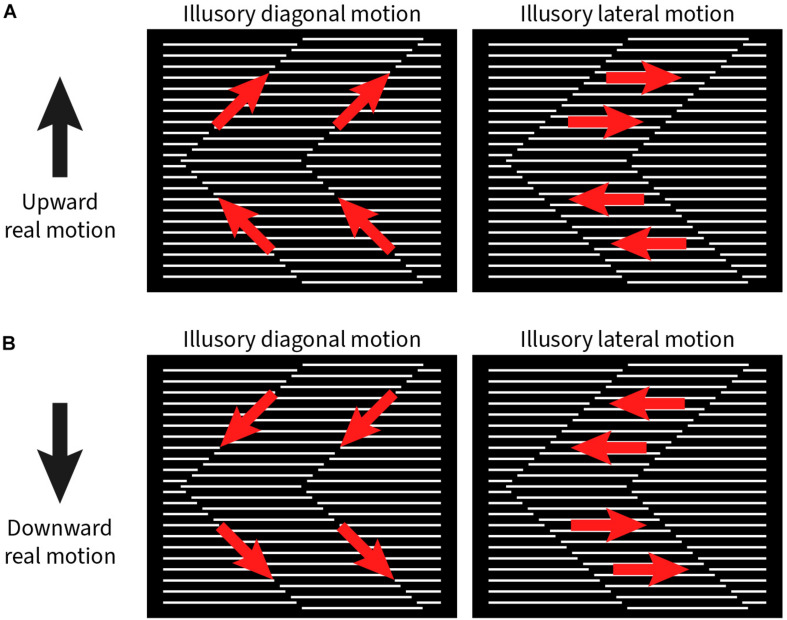
Illustration of illusory motion directions (red arrows) with upward and downward real motion (black arrows) of the stimulus pattern. **(A)** Left panel: Upward real motion produces illusory upward motion along the zigzagging contour between the phase-shifted grating strips. Right panel: In addition, the grating strips appear to move to the right or left, depending on the orientation of the zigzags. **(B)** With downward real motion of the stimulus pattern, the signs for all illusory motion directions are reversed.

Among the six subjects, four exclusively reported diagonal motion along the zigzagging interface between the grating strips (i.e., the path of the illusory contour), while two subjects exclusively reported lateral motion parallel to the line grating. There was little difference in strength for both up and down movement of the stimulus pattern. The relationship between physical and illusory motions are detailed in Figure 2. When the stimulus pattern was moved up, the illusory diagonal motion was also upward along the orientation of the illusory contours (Figure 2A, left); conversely, when the stimulus pattern was moved down, the perceived diagonal motion was perceived along the illusory contours downward (Figure 2B, left). For those subjects who perceived lateral motion, an upward moving stimulus pattern elicited sideward motion to the right for + 45° and left for the −45° grating strips (Figure 2A, right). In the downward moving stimulus pattern, the illusory motion was to the left or right, but swapped (Figure 2B, right).

We also wished to better understand how the absence of the illusory contours between strips affect the perception of illusory motion. First, we slightly interleaved (Supplementary Figure 1B and Supplementary Video 5) or moved apart (Supplementary Figure 1C and Supplementary Video 6) the grating lines, thereby creating an overlap or gap between the abutting endpoints. This abolishes the illusory contour. Second, we superimposed a real contour onto the illusory contour (Supplementary Figure 1D and Supplementary Video 7). In all these variants of the original pattern, reports of illusory motion were abolished for all observers.

#### Conclusion and Discussion

We conclude that two kinds of illusory motion are perceived in the Serpentine Illusion, one parallel to the zigzagging illusory contour (termed diagonal motion); the other parallel to the orientation of the physical line gratings (termed lateral motion). Both kinds of motion can be seen with upward and downward real movement of the stimulus pattern. The direction of the two kinds of illusory motion reverses with the direction of the pattern motion (up or down), while their salience is similar under the two conditions. Readers are invited to play the movie (Supplementary Video 1) that demonstrates the standard stimulus pattern. By locally manipulating the interface that generates the illusory contours, we found that this interface is essential for both kinds of Serpentine Illusory motion.

### Experiment 2. Effect of Stimulus Parameters on Illusory Motion

#### Stimuli

**FIGURE 3:**
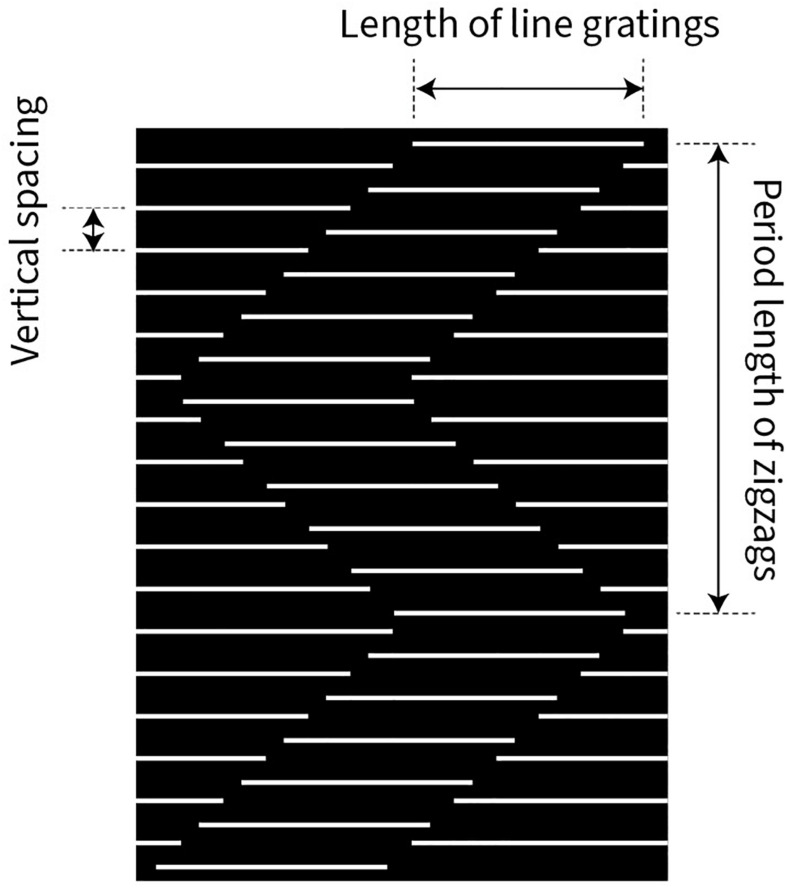
Illustration of three variables, governing the strength of the Serpentine Illusion: vertical spacing between the line gratings, length of the line gratings, and period length of the zigzags (vertical extent). The speed of the stimulus pattern moving upward or downward was also varied but is not shown here.

We used a stimulus pattern similar to that described in Figure 1. Four parameters of the stimulus pattern were quantitatively tested (Figure 3): vertical spacing of the line gratings, length of the line gratings, period length of the zigzags, and speed of up or down stimulus motion.

#### Procedure

**FIGURE 4:**
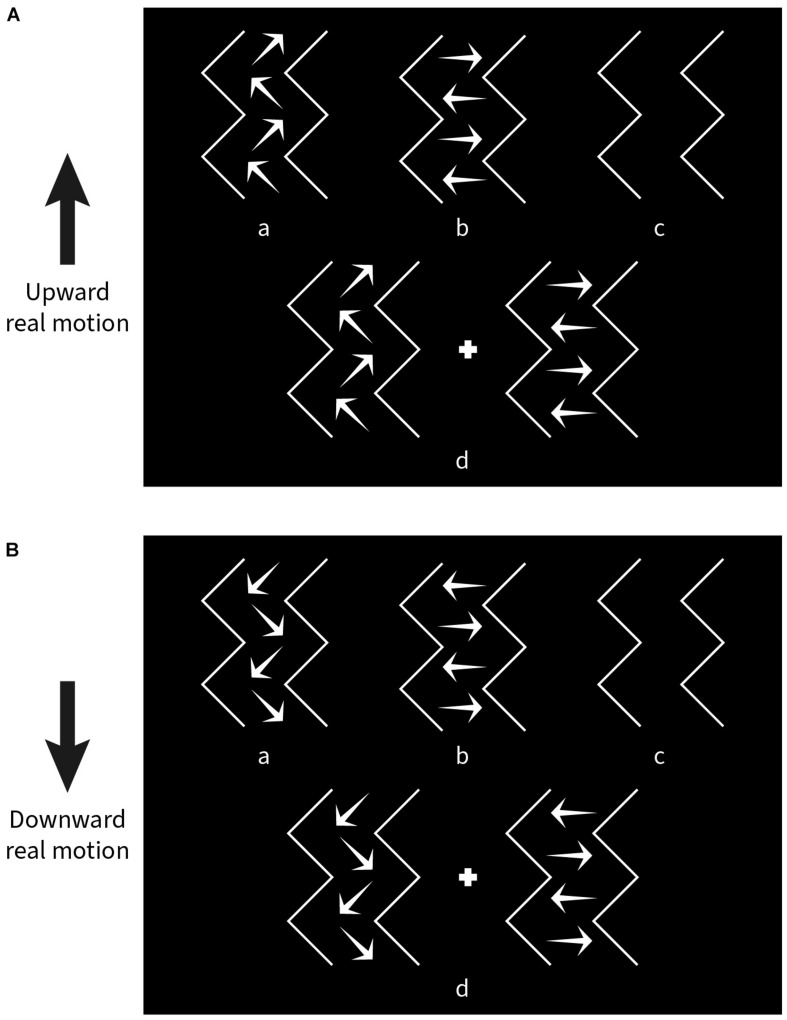
Choice panels used to match the kind of illusory motion perceived for a given experimental condition. **(A)** Response options shown when the stimulus pattern was moved upward. **(B)** Response options shown after downward movement. “a” diagonal motion only, “b” lateral motion only, “c” no illusory motion, “d” both diagonal and lateral motion.

Nine subjects (6 subjects from the previous experiment plus 3 new subjects) participated in the task. The experiment was divided into four blocks; only one parameter was varied in each, while the other parameters were kept the same as in the standard pattern (see Experiment 1). In a given block, there were nine steps for each varied parameter and 10 repetitions for each step. The values of the steps are given in the Results section. The stimulus pattern was moved up or down for each combination of parameters. Altogether, a total of 180 trials (2 × 9 × 10) were randomly presented in each block. Subjects were again instructed to fixate on the central red dot. After stimulus presentation, a panel with four choice pictures containing directional arrows was shown (Figure 4), and subjects were asked to select the picture that most resembled the perceived direction(s) of illusory motion by pressing one of four buttons on the keyboard. The designations of the four choices were as follows:

a) Only diagonal motion (DM) perceived.
b) Only lateral motion (LM) perceived.
c) No illusory motion (NIM) perceived.
d) Both diagonal and lateral motion (BM) perceived.

Two choice panels were designated separately for upward (Figure 4A) and downward (Figure 4B) pattern movement trials.

#### Data Analysis

Relative choice frequencies (in percent) for every single condition were calculated using the following formulas:

(1) $$F_{D\,M}=\;\,\frac{C_{a}}{C_{a}+\;\,C_{b}+\;\,C_{c}+\;\,C_{d}}$$

(2) $$F_{L\,M}=\;\,\frac{C_{b}}{C_{a}+\;\,C_{b}+\;\,C_{c}+\;\,C_{d}}$$

(3) $$F_{B\,M}=\;\,\frac{C_{d}}{C_{a}+\;\,C_{b}+\;\,C_{c}+\;\,C_{d}}$$

where C_*a*_, C_*b*_, C_*c*_ and C_*d*_ are the number of a, b, c and d choices, respectively; *F*_*DM*_ is the relative choice frequency of diagonal motion, *F*_*LM*_ the relative choice frequency of lateral motion, and *F*_*BM*_ the relative choice frequency of both diagonal motion and lateral motion. Choice frequency (in%) is plotted as a function of steps per condition on Cartesian coordinates.

#### Results

**FIGURE 5:**
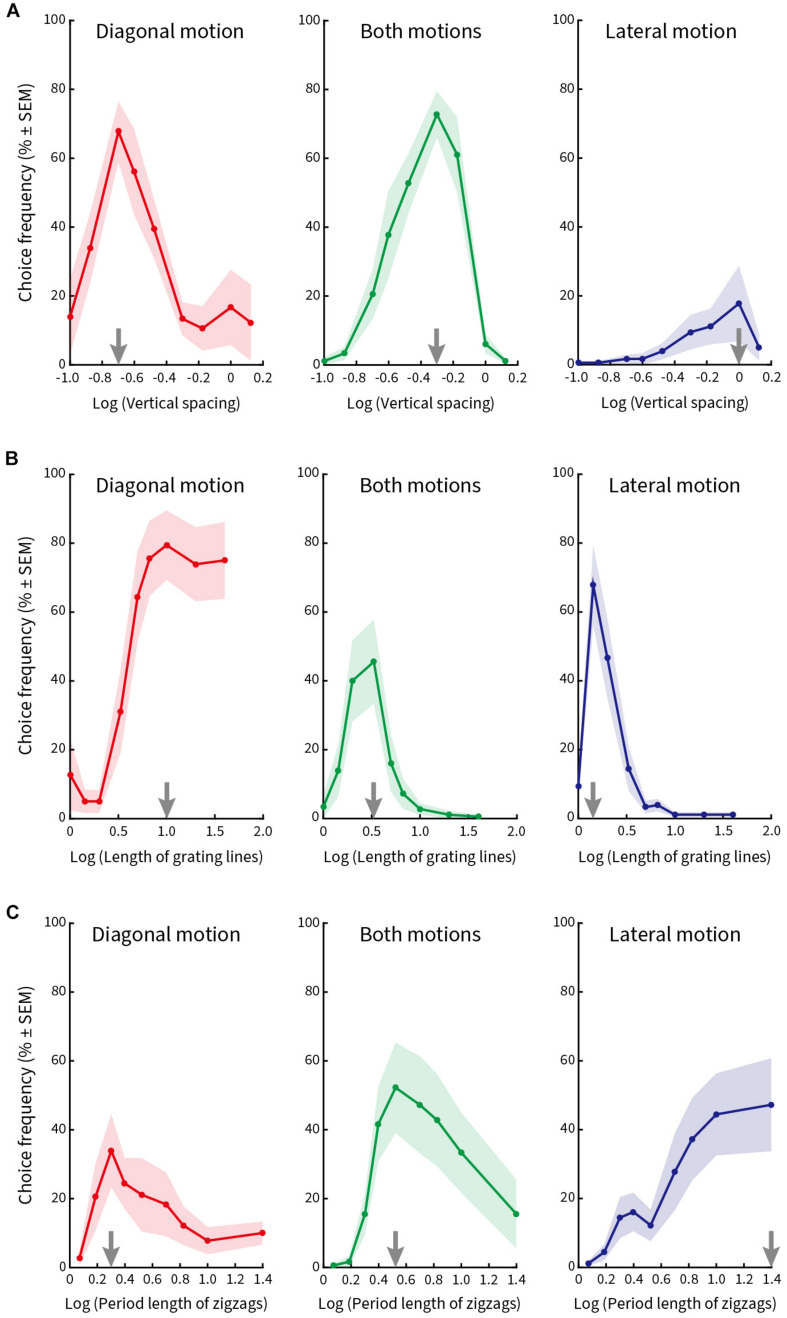
Relative choice frequency plotted as a function of the logarithm of each of three stimulus variables. Curves refer to three kinds of illusory motion perception: diagonal (red), both diagonal and lateral (green), lateral (blue). Each dot on the solid curve represents average responses of nine subjects, shading represents ± 1 SEM. The value at which the curves peak is shown by gray arrows on the abscissa. **(A)** Vertical spacing was varied. Peak choice frequency corresponds to a vertical spacing of 0.2° for diagonal motion, 0.5° for both motions, and 1.0° for lateral motion. **(B)** Length of the line gratings was varied. Peak choice frequencies correspond to line lengths of 10°, 3.3° and 1.4°. **(C)** Length of zigzag period was varied. Peak choice frequencies correspond to lengths of zigzag periods of 2°, 3.3° and 25°, respectively.

In the first block, we varied the vertical spacing of the line gratings in nine steps (0.10°, 0.13°, 0.20°, 0.25°, 0.33°, 0.50°, 0.67°, 1.00°, and 1.33°), while keeping the length of the lines (2.5°), the period length of the zigzags (5°), and the speed of the stimulus pattern (5°/s) constant. Figure 5A plots relative choice frequency on the ordinate as a function of vertical spacing on the abscissa for three kinds of illusory motion perception (diagonal, both diagonal and lateral, lateral). The spacing values were transformed logarithmically to better visualize the distribution of the choice frequencies. The plotted data show the average values from nine subjects (mean ± SEM).

The vertical line spacing at which the individual curves peak is marked by a gray arrow on the abscissa. Subjects chose predominantly diagonal motion when the vertical spacing between the lines was small (Figure 5A, left column); they chose both kinds of motion more frequently when the spacing was intermediate (Figure 5A, middle column); and chose (although less often) lateral motion when the vertical spacing was large (Figure 5A, right column). Therefore, the maximal choice frequency proceeded from a percept of diagonal motion to both diagonal and lateral motion and then lateral motion as the vertical spacing of the line gratings increased from small to large.

In the second block, we varied the length of the line gratings in nine steps (1°, 1.43°, 2°, 3.33°, 5°, 6.67°, 10°, 20°, and 40°), while keeping the vertical line spacing (0.67°), period length of the zigzags (5°), and pattern speed (5°/s) constant. Figure 5B plots relative choice frequency as a function of line length for the same three kinds of illusory motion perception. The peaks of the curves are again illustrated by gray arrows on the abscissa. Subjects designated diagonal motion predominantly when line gratings were long (Figure 5B, left column), a percept of both motions when line length was intermediate (Figure 5B, middle column), and lateral motion when lines were short (Figure 5B, right column).

In the third block, we varied the period length of the zigzags in nine steps (1.18°, 1.54°, 2°, 2.5°, 3.33°, 5°, 6.67°, 10°, and 25°) and kept line spacing (0.67°), length of line gratings (2.5°), and speed of pattern movement (5°/s) constant. Figure 5C plots relative choice frequency as a function of the period length of the zigzags. Gray arrows on the abscissa mark the peak of the individual curves for diagonal motion (Figure 5C, left column), both motions (Figure 5C, middle column), and lateral motion (Figure 5C, right column) as period length of the zigzags increased. We found that subjects perceived diagonal motion at small period lengths, and this transitioned to lateral motion as the period length increased.

**FIGURE 6:**
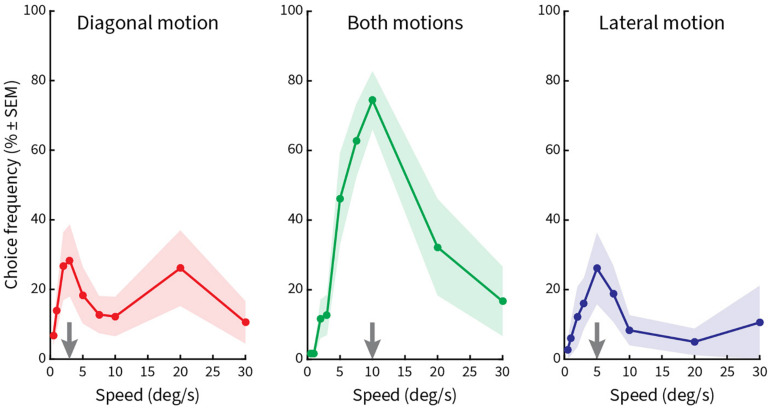
Relative choice frequencies plotted as a function of speed of the stimulus pattern. The layout is the same as in Figure 5. Stimulus pattern speed was varied. A high peak occurs for choice frequencies at 10°/s, with additional low peaks at 3°/s, 5°/s, and 20°/s.

Finally, in the fourth block (Figure 6), pattern speed was varied in nine steps (0.5°/s, 1°/s, 2°/s, 3°/s, 5°/s, 7.5°/s, 10°/s, 20°/s, and 30°/s), while vertical spacing (0.5°), length of line gratings (2.5°), and period length of the zigzags (5°) were kept constant. Here, we found a significant peak only at a speed of 10°/s for both kinds of motions (Figure 6, middle column). Smaller peaks occurred at 3°/s and 20°/s for diagonal motion (Figure 6, left column) and 5°/s for lateral motion (Figure 6, right column). This result implies that illusory motions in the Serpentine Illusion can be seen with all stimulus speeds from 3 to 20°/s, with a maximum at 10°/s.

Based on the above results, we summarized the parametric values, which were found to be optimal for each type of illusory motion perception (Table 1).

**TABLE 1:** Optimal parametric values for each type of illusory motion perception (given in degrees).

Subject responses	Stimulus variables		
	Vertical line spacing	Length of line gratings	Period length of zigzags
Diagonal motion	Narrow (0.33°)	Long (5.00°)	Short (2.00°)
Both motions	Intermediate (0.50°)	Intermediate (2.50°)	Intermediate (5.00°)
Lateral motion	Wide (0.67°)	Short (1.43°)	Long (6.67°)

#### Conclusion and Discussion

In this experiment, we identified the experimental conditions under which each type of illusory motion perception is optimal. For diagonal motion these are: narrow vertical line spacing, long line gratings, and short period of zigzags. For both kinds of motions: intermediate vertical line spacing, intermediate length of line gratings, and intermediate period length of zigzags. For lateral motion: wide vertical line spacing, short line gratings, and long period length of zigzags. The nominal values in degrees of visual angle are given in parentheses in Table 1. As this was a univariate design, there may be subtle interactions between these three major parameters that we were not able to identify.

We constructed three patterns according to the above results and confirmed perceptually that they elicit vivid diagonal motion (Supplementary Figure 2), vivid diagonal and lateral motion (Supplementary Figure 3), and vivid lateral motion (Supplementary Figure 4) as predicted. Readers are invited to play the movies (Supplementary Videos 2–4).

### Experiment 3. Test of Equiluminant Colored Patterns

All patterns studied so far were of high contrast. There is evidence that certain motion illusions weaken or even disappear when their inducing patterns are presented with low or zero luminance contrast (Khang and Essock, 1997; Cavanagh and Anstis, 2002; Hamburger, 2012). In order to test whether the Serpentine Illusion depends on luminance contrast, we systematically varied the luminance contrast between colored line gratings and a colored background. Motion perception is primarily mediated by the magnocellular system, which is thought to be mostly color insensitive. A failure of the Serpentine Illusion to show under equiluminant conditions would therefore confirm a predominant contribution of the magnocellular pathway.

#### Stimuli

Pattern parameters were taken from the both-motions optimized figure (Table 1). Two types of abutting grating patterns were utilized in this experiment, one with red lines on a green background and another with green lines on a red background. The luminance of the background was fixed at 12.10 cd/m^2^, while the luminance of the line gratings was varied in nine steps (0, 2.95, 6, 9.05, 12.10, 15.14, 18.19, 21.24, and 24.29 cd/m^2^). Contrast (C) was defined by using the following equation:

(4) $$C=\frac{L_{g}-L_{b}}{L_{g\,m\,a\,x}}$$

where *L*_*g*_ and *L*_*b*_ represent the luminance of the line gratings and the background, respectively, and *L*_*gmax*_ the highest luminance value set for the line gratings. Depending on the above formula, contrast values range from −0.5 to 0.5, where 0.5 presents the highest luminance of the line gratings and −0.5 the lowest luminance. Physical equiluminance by definition is achieved when both the line gratings and the background have a luminance of 12.10 cd/m^2^, resulting in *C* = 0.

#### Procedure and Data Analysis

Five subjects who participated in experiment 2 took part in this experiment. The procedure was similar to that described in Experiment 2, except that in each condition one type of colored grating pattern was presented. Each experiment block also contained two directions of stimulus motion, 9 luminance contrasts and 10 repetitions, resulting in a total of 180 trials. Choice frequencies (in percent) were calculated according to the following formula:

(5) $$F_{I\,M}=\frac{C_{a}+C_{b}+C_{d}}{C_{a}+C_{b}+C_{c}+C_{d}}$$

where *C*_*a*_, *C*_*b*_, *C*_*c*_ and *C*_*d*_ represent the numbers for choices a, b, c, and d, respectively; *F*_*IM*_ is the relative choice frequency of illusory motion including diagonal, lateral, and both kinds of motion.

#### Results

**FIGURE 7:**
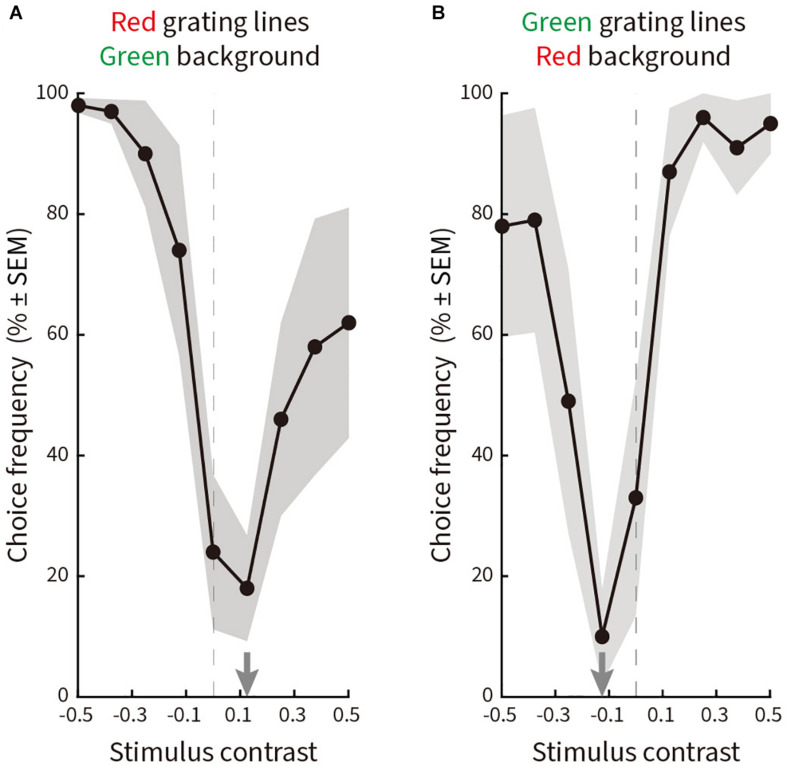
Relative choice frequency plotted as a function of log luminance contrast for line gratings ranging from 0 to 24.29 cd/m^2^ in nine steps on a background of 12.10 cd/m^2^. Solid curves represent averages of a total of 180 choices by five subjects, shading represents ± SEM. The trough of each curve is given by a gray arrow on the abscissa. Equiluminance is indicated by a dashed vertical line. The layout is the same as in Figure 5. **(A)** Red line gratings on a green background. The curve reaches a minimum at a contrast of 0.125 (15.14 cd/m^2^). **(B)** Green line gratings on a red background. The curve reaches a minimum at a contrast of –0.125 (9.05 cd/m^2^).

The resulting curves (Figure 7) plot choice frequency as a function of luminance contrast. Equiluminance is given by the dashed vertical line. Subjects predominantly chose the “No illusion” option (*C*_*c*_) when the line gratings and the background were equiluminant or near-equiluminant, while choosing options *C*_*a*_, *C*_*b*_, or *C*_*d*_ when the luminance contrast increased. The trough of the response curves was shifted slightly to the right (Figure 7A) or to the left (Figure 7B) of physical equiluminance, suggesting a small asymmetry between red-on-green versus green-on-red.

#### Conclusion and Discussion

Results show that the Illusory motion in our patterns was largely eliminated at equiluminance. This is similar to other motion illusions (Khang and Essock, 1997; Cavanagh and Anstis, 2002; Hamburger, 2012). The finding that color contrast alone elicits very weak illusory motion perception suggests a magnocellular dominated origin of the Serpentine Illusion. This distinguishes it somewhat from physical motion, which though degraded can still be perceived in stimuli with pure chromatic contrast (Willis and Anderson, 2002; Takeuchi et al., 2003; McKeefry and Burton, 2009).

### Experiment 4. Effects of Luminance Contrast

Experiment 3 demonstrates that the luminance contrast between colored line gratings and a differently colored background is crucial for generating the Serpentine Illusion. Previous studies using real motion stimuli demonstrate that luminance contrast influences perceived motion (Campbell and Maffei, 1981; Thompson, 1982; Stone and Thompson, 1992; Blakemore and Snowden, 1999; Johnston et al., 1999; Anstis, 2003). In this experiment, we ask how luminance contrast relates to the salience of the Serpentine illusory motion.

#### Stimuli

A stimulus pattern from Table 1 that elicits both diagonal and lateral illusory motion was used. Line spacing, length of line gratings, and period length of zigzags were fixed at values that maximize the “both motions” response (Table 1, row “Both motions”), while the contrast between the line gratings and the background was varied. Both the luminance of the background and the line gratings were varied to generate patterns with Michelson contrasts of 10%, 20%, 40%, 60%, 80% and 100%. Michelson contrast (C_*m*_) was defined as follows:

(6) $$C_{m}=\frac{L_{g}-L_{b}}{L_{g}+L_{b}}$$

where L_*g*_ was the luminance of the line gratings and L_*b*_ the luminance of the background.

#### Procedure and Data Analysis

Five subjects who participated in experiment 2 took part in this experiment. The procedure was similar to that described in Experiment 2, except that contrast was the varied parameter. Choice frequencies (in percent) were calculated using formula (4) which is described in Experiment 3.

#### Results

**FIGURE 8:**
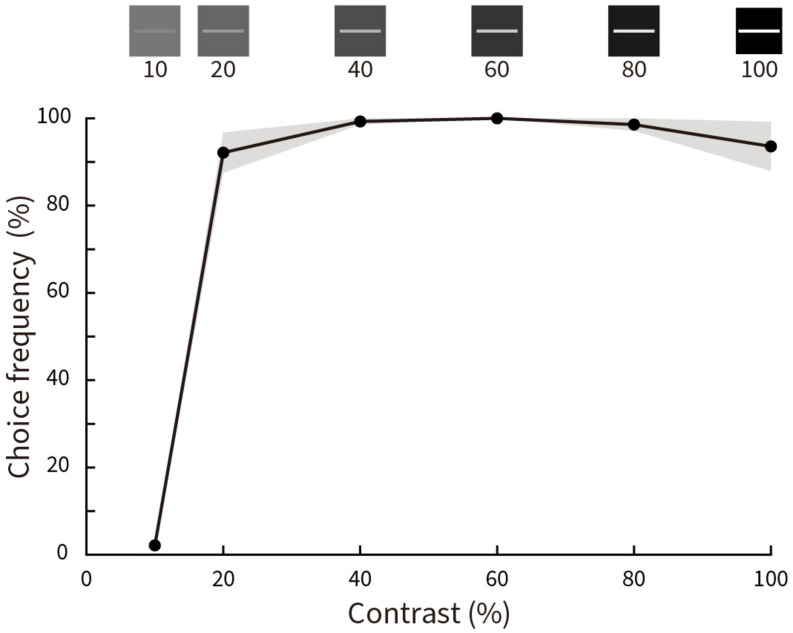
Relative choice frequency plotted as a function of Michelson contrast of the stimuli. Solid curves represent averages of five subjects, shading represents ± SEM. Stimulus contrasts are illustrated at the top. Choice frequency is close to zero at 10% contrast, but almost 100% for all other conditions.

Figure 8 shows the results for choice frequency as a function of luminance contrast. Illusory motion is absent for a luminance contrast of 10%, then rises steeply to almost 100% at a contrast of 20% before leveling off with a subtle, but insignificant peak at 60% (*p* = 0.25 for 20% contrast condition and *p* = 0.5 for 100% contrast condition, Wilcoxon signed rank test).

#### Conclusion and Discussion

We found that the Serpentine Illusion is absent at very low luminance contrast of the stimulus, then acquires full strength at a contrast of 20% and thereafter asymptotes. However, we did not find any significant perceptual difference for contrasts ranging from 20 to 100%, whereas for physical motion there is a progressive increase in motion strength with stimulus contrast (Thompson, 1982; Stone and Thompson, 1992). This difference may point to potentially different coding mechanisms.

### Experiment 5. Illusory Motion Perception After Adaptation

To further investigate the visual stimulus features that may contribute to the illusory motion perception, we used adaptation (Blakemore and Campbell, 1969; Montaser-Kouhsari et al., 2007; Webster, 2015) to different stimulus features of the Serpentine illusion pattern. By extending the presentation time of patterns of static or moving lines/random dots, the specific neuronal circuits representing those features adapt and contribute less to subsequently presented illusory stimuli.

#### Stimuli

**FIGURE 9:**
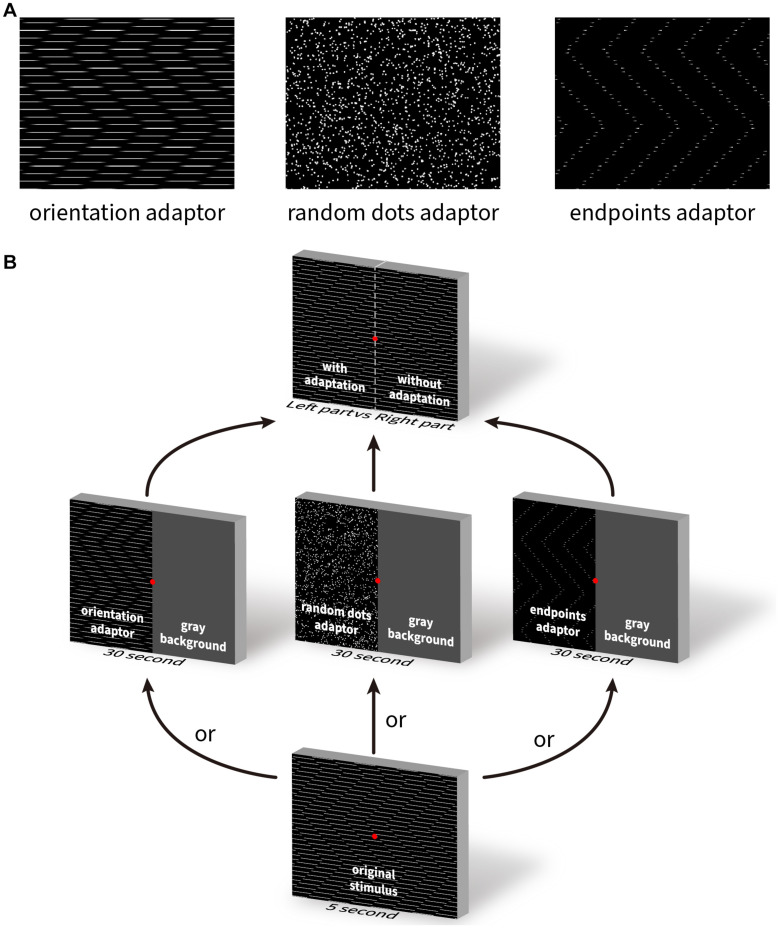
The illustration of three adapting stimuli and the adaptation task paradigm. **(A)** Three visual patterns used as the adapting stimuli. Left: zigzagging line gratings with Gaussian blurred end points; middle: random dots; right: end points only without lines. **(B)** Schematic illustration of the task procedure. The test stimulus was presented for 5 s, followed by the presentation of an adapting stimulus for 30 s, and then again, the testing stimulus for pre- and post-adaptation comparison.

For adapting patterns we used (i) horizontal line gratings presented with the abutting end points eliminated; (ii) random dots with no orientation information, and (iii) end points with only short residual stubs of lines preserved. For the orientation and endpoint adaptors we used the parameters that generate optimal diagonal and lateral illusory motion from Experiment 3 (Table 1). We used gradual alpha-blending (where the opacity of the line changed from opaque in the center to transparent at the edges) at the transition points to reduce introducing abrupt contours where the line or line end was removed. The adapting patterns are shown in Figure 9A. They were presented smoothly moving (upward | downward), or statically flashed at 5 Hz to eliminate directional motion. The motion speed was 5°/s, the density of the random dots was 1 dot/deg^2^, and the diameter of each dot was 0.2°.

#### Procedure

Five experienced subjects participated in the experiment. They were first shown the original Serpentine Illusion pattern from experiment 2 for 5 s. Next, one hemifield was randomly assigned one of the twelve adaptors and the other hemifield was blanked out. After the 30 s of adaptation, the original test pattern was shown again for 5 s. Subjects were asked to report the relative strength between the adapted and non-adapted hemifields for diagonal or lateral motion. This strength was quantified using a scale from 1 to 9, where 1 = no illusory motion, and 9 = full illusory motion (i.e., no effect of adaptation observed). Each of the adapting conditions was repeated 9 times, and all conditions were interleaved and randomly presented during the experiment. Figure 9B illustrates the task procedure.

#### Results

**FIGURE 10:**
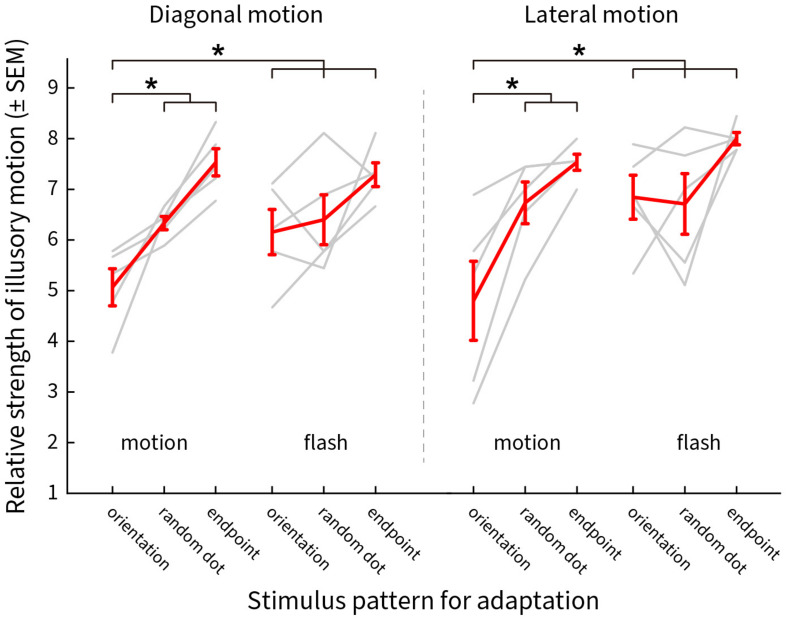
The distribution of the reported scores weighted by the saliency of the illusory motions after adaptation. *Y*-axis shows the mean ± SEM of the scores, *X*-axis presents the adapting stimulus conditions. Results for diagonal and lateral illusory motion are shown on the left and right, respectively. Red lines show the mean values for five subjects, light gray lines show the results for individual subjects. An asterisk (*) denotes a significant difference between the two conditions compared.

The responses for motion and flashed adapting patterns are shown in Figure 10, red representing the group averages and gray for the individual subject scores. The weighted strength of illusory motion is plotted as a function of the stimulus conditions. Adapting to a pattern of smoothly moving line gratings with blurred end points (orientation) produced the lowest scores, implying that this condition had the strongest adaptation effect for both diagonal (Figure 10 left, moving orientation [a1]: 5.07 ± 0.37, moving random dot [a2]: 6.33 ± 0.13, moving endpoint [a3]: 7.53 ± 0.27, flashed orientation [a4]: 6.16 ± 0.45, flashed random dot [a5]: 6.4 ± 0.49, flashed endpoint [a6]: 7.29 ± 0.23, *P*_*a*1–*a*2_ = 0.011, *P*_*a*1–*a*3_ = 2.07 × 10^–8^, *P*_*a*1–*a*4_ = 0.0268, *P*_*a*1–*a*5_ = 0.0025, *P*_*a*1–*a*6_ = 2.1 × 10^–8^) and lateral illusory motion (Figure 10 right, moving orientation [b1]: 4.8 ± 0.78, moving random dot [b2]: 6.73 ± 0.41, moving endpoint [b3]: 7.53 ± 0.16, flash orientation [b4]: 6.84 ± 0.43, flash random dot [b5]: 6.71 ± 0.6, flash endpoint [b6]: 8 ± 0.12, *P*_*b*1–*b*2_ = 0.0023, *P*_*b*1–*b*3_ = 2.07 × 10^–8^, *P*_*b*1–*b*4_ = 2.23 × 10^–5^, *P*_*b*1–*b*5_ = 1.08 × 10^–5^, *P*_*b*1–*b*6_ = 2.07 × 10^–8^).

#### Conclusion and Discussion

The results show that line gratings with blurred end points cause greater adaptation of the mechanisms that generate illusory motion (resulting in statistically weaker illusory motion perception). We also found significant effects of motion compared to static flashing. The weakest effects of adaptation were for chains of end points alone, though this is perhaps expected given the overall pattern difference between this and other stimuli. It was not possible to selectively control for these differences given the spatiotemporal distribution of signals during the motion of the pattern. The results indicate that orientation and motion direction contribute to the Serpentine illusory motion. This implies that serpentine illusory motion cannot be simply explained by lower-level neural coding mechanisms that are predominantly responsible for either direction or orientation alone.

### Experiment 6. Spatiotemporal Energy Model

To better understand the underlying neural mechanisms, we were interested in testing whether model neurons exhibit changes in their direction tuning curves consistent with illusory motion. The spatiotemporal energy model was first proposed by Adelson and Bergen (1985) as an intuitive and compact model of motion processing broadly consistent with area V1 physiology. The model proposes linear filters selective for motion energy within a particular spatiotemporal band that are combined together, and has been used to successfully model many classes of motion patterns (Baker and Issa, 2005; Mante and Carandini, 2005; An et al., 2012; Yin et al., 2015). The model’s Fourier transform of the input image precludes the ability to estimate local spatial position differences, but does estimate responses to the global energy contained in the pattern. We used control stimuli whose illusory contours are orthogonal to the line patterns (Supplementary Figure 5 top row), where only the physical direction of line pattern motion should be observed in the direction tuning curve of modeled neurons. For stimuli that contain a single tilt (Supplementary Figure 5 middle row), if the energy model is consistent with illusory perception, we predict that the tuning curves will shift clockwise or anti-clockwise depending on the combination of tilt and physical motion direction (for details of how predictions are made please refer to Luo et al., 2019). For the zigzag patterns (Supplementary Figure 5 bottom row), both clockwise and anti-clockwise relative illusory motions are present and should therefore cause an increase in bandwidth without an overall shift in preferred direction.

#### Procedure

The equations defining the energy model predictions are adopted from the work of Baker and Issa (2005). Three filters for static visual features are described as Gaussian functions; each specific to spatial frequency, temporal frequency and direction tuning:

(7) $$S\,\left(p\right)=e\,x\,p\,\left\{-\frac{{\left[\log_{2}\left(p\right)-\log_{2}\left(S_{p}\right)\right]}^{2}}{2\,\sigma_{S}^{2}}\right\}$$

(8) $$T\,\left(\rho \right)=e\,x\,p\,\left\{-\frac{{\left[\log_{2}\left(\rho \right)-\log_{2}\left(T_{p}\right)\right]}^{2}}{2\,\sigma_{T}^{2}}\right\}$$

(9) $$\text{Ω}\,\left(\phi \right)=e\,x\,p\,\left[-\frac{{\left(\phi -\phi_{p}\right)}^{2}}{2\,\sigma_{\text{Ω}}^{2}}\right]$$

In these equations, *p* represents the spatial frequency, ρ the temporal frequency, and φ the orientation parameters. S_*p*_, T_*p*_, and φ_*p*_ are the peak spatial frequency, temporal frequency and orientation, respectively. σ_*S*_, σ_*T*_, and σ_Ω_ are proportional to the spatial frequency bandwidth, temporal frequency bandwidth and direction tuning curve bandwidth.

The final response strength of a model neuron to a stimulus is given by:

(10) $$R=\int_{0}^{\infty }\int_{-\pi }^{\pi }\int_{0}^{\infty }A\,\left(p,\phi ,\rho \right)\cdot S\,\left(p\right)\cdot \text{Ω}\,\left(\phi \right)\cdot T\,\left(\rho \right)\,p\,d\,p\,d\,\phi \,d\,\rho$$

*R* is the neural response, *A* is the temporal and spatial Fourier transform of the stimulus. The response is modeled as the linear integration over all spatial and temporal frequencies, and orientations of the stimulus. The parameters for each model neuron were taken from the corresponding real direction selective V1 neuron’s preference.

Three classes of stimuli were used for the model predictions (Supplementary Figure 5): a control pattern that does not generate any illusory motion; a single tilt pattern which generates illusory motion in a single direction away from the physical motion; and finally the standard zigzag pattern that generates illusory motions on either side away from the physical motion.

For statistical comparisons across the population results, we use a Kruskal-Wallis one-way ANOVA, *post hoc* Bonferroni corrected. Differences with *P*-values smaller than 0.05 are considered significant.

#### Results

**FIGURE 11:**
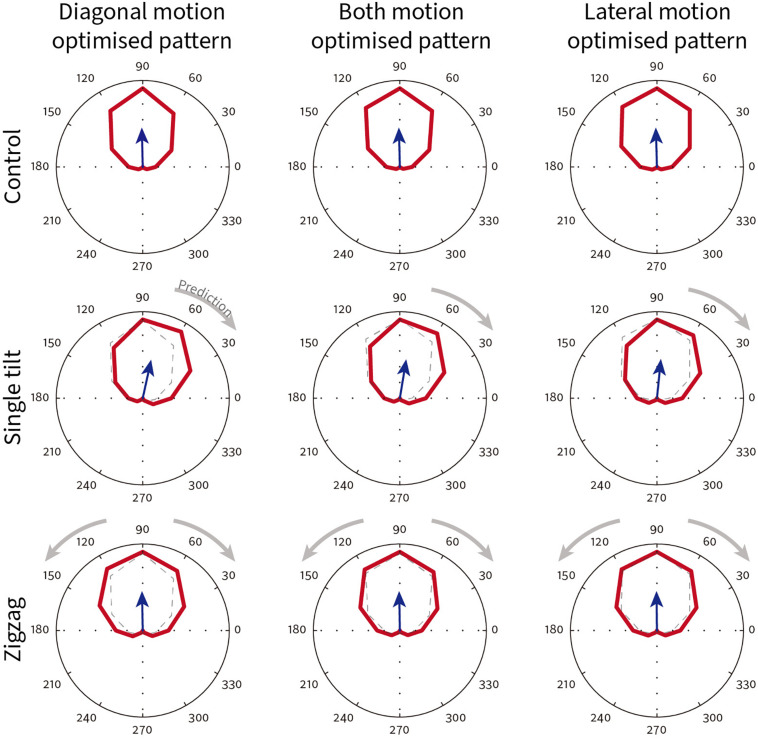
Exemplar model V1 neuron direction tuning curves for different classes of Serpentine illusory motion patterns. This neuron has a preferred direction of 90°. The blue arrow shows the vector summed average direction. Dotted lines in the single-tilt and zigzag conditions reproduce the tuning curve of their respective control condition (top row). Gray arrows show the direction shift predicted from psychophysical observations.

The spatiotemporal energy model parameters (orientation, spatial frequency, temporal frequency) were taken from measurements of 58 V1 direction selective neurons recorded in awake fixating rhesus macaques in our lab. The direction tuning curves for one exemplar model V1 neuron that preferred upward physical motion (90°) is shown in Figure 11. The top row shows the direction tuning responses for control patterns where illusory contours are orthogonal to line orientation. As expected, for all three different types of pattern (diagonal/both motions/lateral), the averaged direction tuning (blue arrow) is 90°. With a single-tilt present in the stimulus pattern (Supplementary Figure 5 middle row), we predicted that there would be a single illusory direction component that should bias the curve clockwise. Such a shift in the preferred direction compared to the control is observed for all three types of illusory motion pattern (Figure 11 middle row; diagonal Δ angle = −13.67°; both Δ angle = −10.76°; lateral Δ angle = −7.43°). For the Serpentine stimulus that contains zigzagged interfaces (Supplementary Figure 5 bottom row), our prediction is that both positive and negative directional shifts relative to physical motion will occur, and this will cause an overall increase in tuning bandwidth. The bottom row of Figure 11 shows that for this exemplar neuron, the bandwidth does increase in all cases (diagonal Δ bandwidth = + 33°; both Δ bandwidth = + 20°; lateral Δ bandwidth = + 16°). One qualitative pattern observed is that diagonal motion optimized patterns show the largest changes in direction.

**FIGURE 12:**
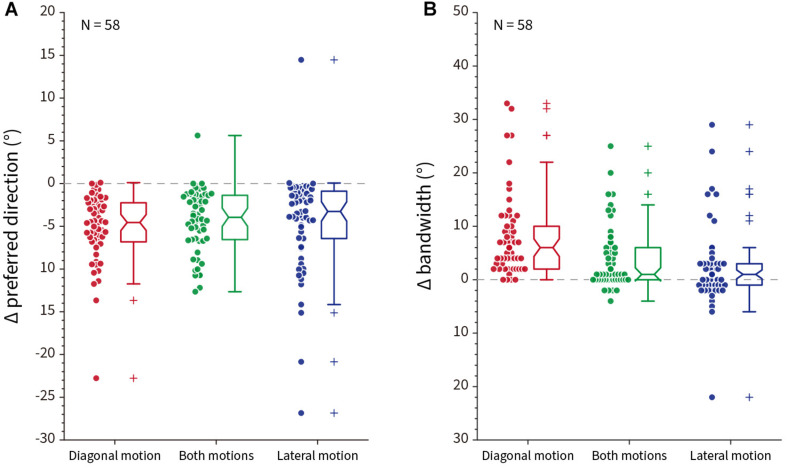
Population response differences compared to control stimuli across all model neurons (*N* = 58). **(A)** The difference (Δ) in preferred direction between control and single-tilt stimulus patterns for diagonal (red), both (green) and lateral (blue) optimized motion stimuli. Notches in the box plot represent the 95% confidence interval of the median. **(B)** The difference (Δ) in tuning curve bandwidth between control and zigzag stimulus patterns, same conventions as **(A)**.

To test whether the effects observed in the exemplar neuron apply across a population of model V1 neurons, we plotted the Δ preferred direction for single-tilt patterns and Δ bandwidth for zigzag patterns across all 58 model V1 neurons. Figure 12 shows combined scatter/boxplots for the (Figure 12A) single-tilt and (Figure 12B) zigzag patterns across the three optimized illusory motion stimuli. Across all model V1 neurons, there is an overall significant predicted preferred direction shift for single-tilt diagonal (−5.17 ± 0.53 SEM, *p* = 2.82 × 10^–20^), both (−4.30 ± 0.48 SEM, *p* = 7.39 × 10^–17^), and lateral (−4.46 ± 0.77 SEM, *p* = 1.51 × 10^–14^) optimized motion stimuli. For zigzag stimuli, there are significant predicted increases in bandwidth for diagonal (7.95 ± 1.00 SEM, *p* = 3.65 × 10^–17^), both (3.93 ± 0.77 SEM, *p* = 5.88 × 10^–6^), but not lateral (2.12 ± 0.96 SEM, *p* = 0.09) optimized motion stimuli.

#### Conclusion and Discussion

From our human psychophysical results (experiment 2), we could make predictions about how the tuning curve biases of V1 cells should respond to different patterns of Serpentine illusory motion patterns. Using a well-established spatiotemporal energy model of V1 neurons, we found that the changes in preferred direction and bandwidth for almost all model neurons were consistent with global predictions. Over the population, diagonal-motion optimized responses showed the largest changes for both preferred direction and bandwidth. If we were to take a simplistic vector sum, lateral motion should generate a bigger directional shift, as lateral illusory motion is orthogonal (−90°) to physical motion. It is hard for us to estimate the relative strength between diagonal and lateral illusory motions subjectively, and we cannot therefore differentiate between the alternative hypotheses that lateral motion should indeed be weaker, or simply that the spatiotemporal energy model provides worse explanatory power for lateral illusory motion. This latter hypothesis could be due to either spatial frequency differences among the three patterns, or some non-linear mechanisms that are necessary for “grouping” the lateral motion across the strip. It is also worth noting that for both-motions optimized stimuli, there was no “additive” effect for combining diagonal and lateral illusory motion together. We do not wish to suggest that we can infer directly from single unit responses to global illusory perception, but previous work has shown that biases present in neurons in early visual areas can be combined downstream in MT and MST to form global perceptions of illusory motion (Luo et al., 2019).

## General Discussion

In this paper, we describe a visual motion illusion, which we have called the Serpentine Illusion. It is elicited by a pattern of phase-shifted grating strips, abutting each other along a zigzagging interface. When the stimulus pattern is moved up or down, the intersections formed by the offset line gratings are seen to move in an undulating snake-like fashion. In addition to this motion along the diagonals, lateral motion of the sections is also seen. The strength of both illusory motions depends on the stimulus parameters. The illusion is luminance-contrast dependent, suggesting that magnocellular pathway signals have a predominant impact on the Serpentine Illusion. Results from selective adaptation show that both line gratings and physical motion are necessary for the full perception of the illusion, and modeling suggests the illusory motion can partly be explained by linear spatiotemporal receptive fields of motion sensitive V1 cells.

### Visual Features Contributing to the Serpentine Illusion

We used an adaptation paradigm to test the contributions of orientation and motion mechanisms. We used moving and flashed grating-lines or grating-endpoints, and random dots, to adapt out the orientation, motion and end-stopping signals driven by the illusion inducing pattern. According to Figure 10, it was the moving line gratings that had the largest effect of weakening the perception of the Serpentine Illusion. To further test the contribution of line gratings for inducing the illusory motions, we introduced a pattern in which the line gratings of the original pattern were occluded by zigzag masks of varying thickness (Supplementary Figure 6). We found that illusory motion was abolished in the pattern with only endpoints visible (Supplementary Figure 6A), and only slightly restored when 1/2 or 1/3 of the grating lines were masked (Supplementary Figures 6B,C); illusory motion was weakened even with a very thin occluding mask (Supplementary Figure 6D). These observations suggest that continuous line gratings are crucial for the generation of both diagonal and lateral illusory motions.

### Differences and Similarities Between Lateral and Diagonal Motion

Diagonal motion occurs primarily when the local contrast differences driven by the endpoints follow each other at close range (i.e., high density). This is the case when the line gratings are narrowly spaced, when the distance between the end points of a line is long and when the period length of the zigzags is short. This will make a grating strip look like an undulating column. On the other hand, lateral motion is favored by low density chains of end points as found with widely spaced line gratings, short horizontal distances between pairs of end points, and a long period of zigzags; these features favor a percept of horizontally arranged rows of end points moving sideward together. Apart from the parameters tested in Experiment 2, we further varied the angle of the zigzagging abutting interface. When the angle is changed to ± 30°, illusory motion along the diagonals predominates (Supplementary Figure 7A), whereas when the angle is changed to ± 60°, strong lateral motion is perceived (Supplementary Figure 7B). In the former case, the zigzags emphasize the vertical columnar structure, whereas at more acute angles the columns are less salient and the horizontal structure predominates. These parametric conditions for seeing diagonal and lateral illusory motion produce groupings that are consistent with the Gestalt principles of proximity and common fate. Whether these two illusory motion patterns drive the same or different underlying neural mechanisms remains unknown. The fact that either of the motion patterns can be almost eliminated when optimizing for the other condition (Supplementary Figures 2, 4), suggests the neural mechanisms may be dissociable. Future studies will need to systemically explore the neural origins of both illusory motions using theoretical, psychophysical and physiological methods.

### Illusory Motions Are Luminance-Contrast Dependent

Motion and color signals were classically thought to be encoded differentially (Ramachandran and Gregory, 1978; Zeki, 1978; Livingstone and Hubel, 1988). This parallel division receives some support from psychophysical studies in which chromatic gratings without luminance contrast can effectively weaken the ability of a subject to discriminate motion direction/speed (Cavanagh et al., 1984; Troscianko and Fahle, 1988; Cavanagh and Anstis, 1991; Kooi and Devalois, 1992; Mullen and Boulton, 1992a, b; Henning and Derrington, 1994). Other studies, however, show that equiluminant color contrast can also provide weak cues for motion perception (Cavanagh and Favreau, 1985; Saito et al., 1989; Hawken et al., 1994; Gegenfurtner and Hawken, 1995, 1996a,b; Burr et al., 1998; Dougherty et al., 1999; Lu et al., 1999; Yoshizawa et al., 2000; Willis and Anderson, 2002; Cropper and Wuerger, 2005; Burton and McKeefry, 2007). Cortical areas like MT (Saito et al., 1989; Seidemann et al., 1999; Thiele et al., 1999; Wandell et al., 1999; Barberini et al., 2005) and V3A (McKeefry et al., 2010) are able to encode motion signals derived from chromatically defined stimuli. In addition it is well known that area V4 encodes motion information and contains mixed parvocellular and magnocellular inputs (Desimone and Schein, 1987; Mountcastle et al., 1987; Ferrera and Maunsell, 2005; Tolias et al., 2005; Mysore et al., 2008; An et al., 2012; Li et al., 2013; Yin et al., 2015; Birman and Gardner, 2018). This physiological and anatomical data is consistent with the psychophysical data suggesting physical motion signals are processed through mixed pathways (Willis and Anderson, 2002; Takeuchi et al., 2003; McKeefry and Burton, 2009). Although the exact balance of interactions across form, color and motion signaling circuits is still a matter of some debate, there is nevertheless psychophysical evidence showing that motion illusions are minimized when presented under chromatic equiluminant conditions (Khang and Essock, 1997; Hamburger, 2012).

The original Serpentine Illusion stimulus pattern has high luminance contrast between line gratings and background. When we reduced the contrast to physical equiluminance, illusory motion was greatly weakened (Figure 7). This suggests that luminance contrast is an important factor in generating the Serpentine Illusion, an observation consistent with several other motion illusions, which are found to be luminance-contrast dependent (Hamburger, 2012). Examples include the Pinna-Brelstaff illusion (Pinna and Brelstaff, 2000), the Rotating Snakes illusion (Kitaoka and Ashida, 2003), the Rotating-Tilted-Lines illusion (Gori and Hamburger, 2006), the Boogie-Woogie illusion (Cavanagh and Anstis, 2002), and the Dotted Lines illusion (Ito et al., 2009). This indicates that unlike physical motion, illusory motions in the Serpentine and other motion illusions are largely mediated by the magnocellular pathway. Additionally, previous psychophysical studies found that subjects underestimate the speed of moving gratings with relatively low luminance contrast (Campbell and Maffei, 1981; Thompson, 1982; Stone and Thompson, 1992; Blakemore and Snowden, 1999; Johnston et al., 1999; Anstis, 2003). Analogously, the saliency of illusory motion can also be controlled through changing the strength of luminance contrast in the physical stimulus (Cavanagh and Anstis, 2002; Anstis, 2004; Backus and Oruc, 2005; Howe et al., 2006; Hamburger, 2012). It has been hypothesized that a moderate luminance contrast is the main factor in generating some motion illusions (Foster and Altschuler, 2001; Cavanagh and Anstis, 2002; Conway et al., 2005; Ito et al., 2009; Hamburger, 2012). However, for the Serpentine Illusion, only the extreme low luminance contrast (10% in Figure 8) can reduce the strength of the perceptual illusory motions.

The observations in Experiment 2 point toward a role for local contrast differences between the abutting line endings. Each pair of juxtaposed, phase-shifted line ends produces a dark patch that stands out when the stimulus pattern is moved up or down. One possible explanation of the illusory motion seen is due to the larger contrast of the dark patch relative to the grating strips. It is known that high-contrast gratings appear to move faster than low-contrast gratings (Thompson, 1982; Stone and Thompson, 1992) and this might explain the faster rate at which the dark patches are seen to be moving relative to the flanking line gratings.

### Alternatives to the Energy Model

Without directly recording from the nervous system, theoretical models are one of the best ways to infer neural mechanisms that underlie visual perceptual phenomenon. Here, we used the classical motion energy model (Adelson and Bergen, 1985) to show that primary visual cortex neurons respond with directional biases consistent with perception to the illusory motions in Serpentine Illusion stimuli. The motion energy model is a simplified linear summation model (Baker and Issa, 2005; Mante and Carandini, 2005), best at predicting response properties in the earliest motion processing stages (Reid et al., 1987; Carandini et al., 1997). Complex cells in primary visual cortex and neurons in downstream visual areas like MT and MST have more non-linear response properties (Emerson et al., 1992; Simoncelli and Heeger, 1998; Pack et al., 2006), and they contribute significantly to the neural representation and perception of illusory motions (Luo et al., 2019). Future studies should explore compare both linear and non-linear coding components and hierarchical population responses (mirroring the hierarchical spatiotemporal integration of motion information), as these may better predict the cortical responses to Serpentine and other illusory motion patterns.

### Similarities and Differences Between Serpentine and Other Visual Illusions

The diagonal illusory motion along the zigzagging contour of the interface is reminiscent of the apparent motion in other two well-known motion illusions: the Boogie-Woogie illusion (Cavanagh and Anstis, 2002) and the dotted lines illusion (Ito et al., 2009). The Boogie-Woogie illusion is constructed by a grid of horizontal and vertical bars made up from alternating dark and bright squares placed on a gray background. When this grid is moved diagonally from the lower left to the upper right, the small squares making up the bars appear to “race” up the verticals and to the right along the horizontals. The authors attribute their illusion to different strengths of the motion signals elicited by the vertical and horizontal bars. Apparent upward motion resulting from the alternating light and dark edges of the squares moving “along” the bar (first-order motion) is said to constitute a more efficient motion signal than the “across” motion by the textured edge of the horizontal bar which therefore would appear to move more slowly and be overtaken by the vertically moving squares. The dotted lines illusion contains obliquely aligned white and black dots on a median gray background. Horizontal movement of the patten produce relative motion along the row of dots. The authors suggest that illusory motion is produced by the stronger luminance contrast between adjacent dots along the length of the line, compared to with the luminance difference between dotted lines and background.

Interestingly, another illusory motion effect can be perceived when the Serpentine Illusion picture is moved horizontally along the abutting line gratings (or by tracking the cursor as it moves leftward or rightward). The tilted zigzag illusory contours appears to swell out- or inward to each other. Such illusory motions are inconsistent with the aperture effect (Nakayama and Silverman, 1988), and appear related to the dotted-line illusion. Despite similarities, the lateral motion is not observed in either Boogie-Woogie illusion or dotted lines illusion. Moreover, the boogie-woogie illusion elicits the strongest motion at low contrast while the Serpentine Illusion becomes invisible at low contrast. Compared with the Boogie-Woogie and dotted lines illusion, the Serpentine Illusion does not contain luminance contrast along line gratings. Nevertheless, we cannot rule out the possibility that these three illusions may share similar underlying coding mechanisms, since luminance contrast is critical for producing illusory motion in all three patterns, and under equiluminant conditions, illusory motion is largely eliminated in all three patterns. Further psychophysical and physiological experiments are therefore needed to reveal the neuronal mechanisms underlying the Serpentine Illusion.

## Data Availability Statement

The raw data supporting the conclusions of this article will be made available by the authors, without undue reservation.

## Ethics Statement

The studies involving human participants were reviewed and approved by the Ethics Committee of the Institute of Neuroscience, Chinese Academy of Sciences. Subjects gave their consent to the procedure and institutional guidelines. This work adheres to the tenets of the Declaration of Helsinki. The patients/participants provided their written informed consent to participate in this study.

## Author Contributions

WW made the initial phenomenological observation of the illusion. JL, ZC, LS, IA, and WW designed the experiments. JL and ZC performed the experiments and performed data analysis. YL developed the stimulus presentation and energy model code. JL, ZC, LS, and IA wrote the manuscript. All authors contributed to the article and approved the submitted version.

## Conflict of Interest

The authors declare that the research was conducted in the absence of any commercial or financial relationships that could be construed as a potential conflict of interest.

## References

[B1] Adelson E. H. Bergen J. R. (1985). Spatiotemporal energy models for the perception of motion. *J. Opt. Soc. Am. A* 2 284–299. 10.1364/josaa.2.000284 3973762

[B2] An X. Gong H. Qian L. Wang X. Pan Y. Zhang X. (2012). Distinct functional organizations for processing different motion signals in V1, V2, and V4 of macaque. *J. Neurosci.* 32 13363–13379. 10.1523/jneurosci.1900-12.2012 23015427 PMC6621371

[B3] Anstis S. (2003). Moving objects appear to slow down at low contrasts. *Neural Netw.* 16 933–938. 10.1016/s0893-6080(03)00111-4 12850053

[B4] Anstis S. (2004). Factors affecting footsteps: contrast can change the apparent speed, amplitude and direction of motion. *Vision Res.* 44 2171–2178. 10.1016/j.visres.2004.03.015 15183684

[B5] Backus B. T. Oruc I. (2005). Illusory motion from change over time in the response to contrast and luminance. *J. Vis.* 5 1055–1069. 16441202 10.1167/5.11.10

[B6] Baker T. Issa N. (2005). Cortical maps of separable tuning properties predict population responses to complex visual stimuli. *J. Neurophysiol.* 94 775–787. 10.1152/jn.01093.2004 15758052

[B7] Barberini C. L. Cohen M. R. Wandell B. A. Newsome W. T. (2005). Cone signal interactions in direction-selective neurons in the middle temporal visual area (MT). *J. Vis.* 5 603–621. 16231996 10.1167/5.7.1

[B8] Birman D. Gardner J. L. (2018). A quantitative framework for motion visibility in human cortex. *J. Neurophysiol.* 120 1824–1839. 10.1152/jn.00433.2018 29995608

[B9] Blakemore C. Campbell F. W. (1969). On the existence of neurones in the human visual system selectively sensitive to the orientation and size of retinal images. *J. Physiol. Lond.* 203 237–260. 10.1113/jphysiol.1969.sp008862 5821879 PMC1351526

[B10] Blakemore M. R. Snowden R. J. (1999). The effect of contrast upon perceived speed: a general phenomenon? *Perception* 28 33–48. 10.1068/p2722 10627851

[B11] Burr D. C. Fiorentini A. Morrone C. (1998). Reaction time to motion onset of luminance and chromatic gratings is determined by perceived speed. *Vision Res.* 38 3681–3690. 10.1016/s0042-6989(98)00056-x 9893799

[B12] Burton M. P. McKeefry D. J. (2007). Misperceptions of speed for chromatic and luminance grating stimuli. *Vision Res.* 47 1504–1517. 10.1016/j.visres.2006.12.020 17395238

[B13] Campbell F. W. Maffei L. (1981). The influence of spatial frequency and contrast on the perception of moving patterns. *Vision Res.* 21 713–721. 10.1016/0042-6989(81)90080-8 7293002

[B14] Carandini M. Heeger D. J. Movshon J. A. (1997). Linearity and normalization in simple cells of the macaque primary visual cortex. *J. Neurosci.* 17 8621–8644. 10.1523/jneurosci.17-21-08621.1997 9334433 PMC6573724

[B15] Cavanagh P. Anstis S. (1991). The contribution of color to motion in normal and color-deficient observers. *Vision Res.* 31 2109–2148. 10.1016/0042-6989(91)90169-6 1771796

[B16] Cavanagh P. Anstis S. (2002). The boogie-woogie illusion. *Perception* 31 1005–1011. 10.1068/p3378 12269582

[B17] Cavanagh P. Favreau O. E. (1985). Color and luminance share a common motion pathway. *Vision Res.* 25 1595–1601. 10.1016/0042-6989(85)90129-4 3832582

[B18] Cavanagh P. Tyler C. W. Favreau O. E. (1984). Perceived velocity of moving chromatic gratings. *J. Opt. Soc. Am. A* 1 893–899. 10.1364/josaa.1.000893 6470841

[B19] Cohen A. Buia C. Tiesinga P. (2014). Dependence of V2 illusory contour response on V1 cell properties and topographic organization. *Biol. Cybern.* 108 337–354. 10.1007/s00422-014-0602-x 24801874

[B20] Conway B. R. Kitaoka A. Yazdanbakhsh A. Pack C. C. Livingstone M. S. (2005). Neural basis for a powerful static motion illusion. *J. Neurosci.* 25 5651–5656. 10.1523/jneurosci.1084-05.2005 15944393 PMC1431688

[B21] Cropper S. J. Wuerger S. M. (2005). The perception of motion in chromatic stimuli. *Behav. Cogn. Neurosci. Rev.* 4 192–217. 10.1177/1534582305285120 16510893

[B22] Desimone R. Schein S. (1987). Visual properties of neurons in area V4 of the macaque: sensitivity to stimulus form. *J. Neurophysiol.* 57 835–868. 10.1152/jn.1987.57.3.835 3559704

[B23] Dougherty R. F. Press W. A. Wandell B. A. (1999). Perceived speed of colored stimuli. *Neuron* 24 893–899. 10.1016/s0896-6273(00)81036-3 10624952

[B24] Emerson R. C. Bergen J. R. Adelson E. H. (1992). Directionally selective complex cells and the computation of motion energy in cat visual cortex. *Vision Res.* 32 203–218. 10.1016/0042-6989(92)90130-b 1574836

[B25] Ferrera V. Maunsell J. (2005). Motion processing in macaque V4. *Nat. Neurosci.* 8:1125. 10.1038/nn0905-1125a 16127440

[B26] Foster C. Altschuler E. L. (2001). Last but not least: the bulging grid. *Perception* 30 393–395. 11374207 10.1068/p3003no

[B27] Gegenfurtner K. R. Hawken M. J. (1995). Temporal and chromatic properties of motion mechanism. *Vision Res.* 35 1547–1563. 10.1016/0042-6989(94)00264-m 7667913

[B28] Gegenfurtner K. R. Hawken M. J. (1996a). Interaction of motion and color in the visual pathways. *Trends Neurosci.* 19 394–401. 10.1016/s0166-2236(96)10036-9 8873357

[B29] Gegenfurtner K. R. Hawken M. J. (1996b). Perceived velocity of luminance, chromatic and non-Fourier stimuli: influence of contrast and temporal frequency. *Vision Res.* 36 1281–1290. 10.1016/0042-6989(95)00198-0 8711907

[B30] Gori S. Hamburger K. (2006). A new motion illusion: the Rotating-Tilted-Lines illusion. *Perception* 35 853–857. 10.1068/p5531 16836050

[B31] Halko M. A. Mingolla E. Somers D. C. (2008). Multiple mechanisms of illusory contour perception. *J. Vis.* 8 17.1–17.17. 10.1167/8.11.17 18831611

[B32] Hamburger K. (2012). Still motion? Motion illusions and luminance contrast. *Perception* 41 113–116. 10.1068/p7005 22611668

[B33] Hawken M. J. Gegenfurtner K. R. Tang C. (1994). Contrast dependence of color and luminance motion mechanisms in human vision. *Nature* 367 268–270. 10.1038/367268a0 8121491

[B34] Henning G. B. Derrington A. M. (1994). Speed, spatial-frequency, and temporal-frequency comparisons in luminance and color gratings. *Vision Res.* 34 2093–2101. 10.1016/0042-6989(94)90319-0 7941407

[B35] Howe P. D. L. Thompson P. G. Anstis S. M. Sagreiya H. Livingstone M. S. (2006). Explaining the footsteps, belly dancer, Wenceslas, and kickback illusions. *J. Vis.* 6 1396–1405. 17209742 10.1167/6.12.5 PMC2637218

[B36] Ito H. Anstis S. Cavanagh P. (2009). Illusory movement of dotted lines. *Perception* 38 1405–1409. 10.1068/p6383 19911636 PMC5047278

[B37] Johnston A. Benton C. P. Morgan M. J. (1999). Concurrent measurement of perceived speed and speed discrimination threshold using the method of single stimuli. *Vision Res.* 39 3849–3854. 10.1016/s0042-6989(99)00103-0 10748920

[B38] Kanizsa G. (1974). Contours without gradients or cognitive contours? *Ital. J. Psychol.* 1 93–113.

[B39] Kanizsa G. (1976). Subjective contours. *Sci. Am.* 234 48–52. 10.1038/scientificamerican0476-48 1257734

[B40] Kanizsa G. (1979). *Organization in Vision: Essays on Gestalt Perception.* New York, NY: Praeger.

[B41] Khang B. G. Essock E. A. (1997). Apparent relative motion from a checkerboard surround. *Perception* 26 831–846. 10.1068/p260831 9509137

[B42] Kitaoka A. Ashida H. (2003). Phenomenal characteristics of the peripheral drift illusion. *Vision* 15 261–262.

[B43] Kleiner M. Brainard D. Pelli D. Ingling A. Murray R. Broussard C. (2007). What’s new in psychtoolbox-3. *Perception* 36 1–16.

[B44] Kooi F. L. Devalois K. K. (1992). The role of color in the motion system. *Vision Res.* 32 657–668. 10.1016/0042-6989(92)90182-i 1413550

[B45] Li P. Zhu S. Chen M. Han C. Xu H. Hu J. (2013). A motion direction preference map in monkey V4. *Neuron* 78 376–388. 10.1016/j.neuron.2013.02.024 23622068

[B46] Livingstone M. Hubel D. (1988). Segregation of form, color, movement, and depth - anatomy, physiology, and perception. *Science* 240 740–749. 10.1126/science.3283936 3283936

[B47] Lu Z. L. Lesmes L. A. Sperling G. (1999). The mechanism of isoluminant chromatic motion perception. *Proc. Natl. Acad. Sci. U.S.A.* 96 8289–8294. 10.1073/pnas.96.14.8289 10393987 PMC22227

[B48] Luo J. He K. Andolina I. M. Li X. Yin J. Chen Z. (2019). Going with the flow: the neural mechanisms underlying illusions of complex-flow motion. *J. Neurosci.* 39 2664–2685. 10.1523/jneurosci.2112-18.2019 30777886 PMC6445985

[B49] Mante V. Carandini M. (2005). Mapping of stimulus energy in primary visual cortex. *J. Neurophysiol.* 94 788–798. 10.1152/jn.01094.2004 15758051

[B50] McKeefry D. J. Burton M. P. (2009). The perception of speed based on L-M and S-(L plus M) cone opponent processing. *Vision Res.* 49 870–876. 10.1016/j.visres.2009.03.004 19285523

[B51] McKeefry D. J. Burton M. P. Morland A. B. (2010). The contribution of human cortical area V3A to the perception of chromatic motion: a transcranial magnetic stimulation study. *Eur. J. Neurosci.* 31 575–584. 10.1111/j.1460-9568.2010.07095.x 20105228

[B52] Montaser-Kouhsari L. Landy M. S. Heeger D. J. Larsson J. (2007). Orientation-selective adaptation to illusory contours in human visual cortex. *J. Neurosci.* 27 2186–2195. 10.1523/jneurosci.4173-06.2007 17329415 PMC2728022

[B53] Mountcastle V. B. Motter B. C. Steinmetz M. A. Sestokas A. K. (1987). Common and differential effects of attentive fixation on the excitability of parietal and prestriate (V4) cortical visual neurons in the macaque monkey. *J. Neurosci.* 7 2239–2255. 10.1523/jneurosci.07-07-02239.1987 3612240 PMC6568950

[B54] Mullen K. T. Boulton J. C. (1992a). Absence of smooth motion perception in color-vision. *Vision Res.* 32 483–488. 10.1016/0042-6989(92)90240-j 1604835

[B55] Mullen K. T. Boulton J. C. (1992b). Interactions between color and luminance contrast in the perception of motion. *Ophthalmic Physiol. Opt.* 12 201–205. 10.1111/j.1475-1313.1992.tb00290.x 1408173

[B56] Murray M. M. Herrmann C. S. (2013). Illusory contours: a window onto the neurophysiology of constructing perception. *Trends Cogn. Sci.* 17 471–481. 10.1016/j.tics.2013.07.004 23928336

[B57] Mysore S. Vogels R. Raiguel S. Orban G. (2008). Shape selectivity for camouflage-breaking dynamic stimuli in dorsal V4 neurons. *Cereb. Cortex* 18 1429–1443. 10.1093/cercor/bhm176 17934186

[B58] Nakayama K. Silverman G. H. (1988). The aperture problem–I. Perception of nonrigidity and motion direction in translating sinusoidal lines. *Vision Res.* 28 739–746. 10.1016/0042-6989(88)90052-1 3227650

[B59] Pack C. C. Conway B. R. Born R. T. Livingstone M. S. (2006). Spatiotemporal structure of nonlinear subunits in macaque visual cortex. *J. Neurosci.* 26 893–907. 10.1523/jneurosci.3226-05.2006 16421309 PMC1413500

[B60] Pinna B. Brelstaff G. J. (2000). A new visual illusion of relative motion. *Vision Res.* 40 2091–2096. 10.1016/s0042-6989(00)00072-9 10878270

[B61] Ramachandran V. S. Gregory R. L. (1978). Does color provide an input to human motion perception. *Nature* 275 55–56. 10.1038/275055a0 683341

[B62] Ramsden B. M. Hung C. P. Roe A. W. (2001). Real and illusory contour processing in area V1 of the primate: a cortical balancing act. *Cereb. Cortex* 11 648–665. 10.1093/cercor/11.7.648 11415967

[B63] Reid R. C. Soodak R. E. Shapley R. M. (1987). Linear mechanisms of directional selectivity in simple cells of cat striate cortex. *Proc. Natl. Acad. Sci. U.S.A.* 84 8740–8744. 10.1073/pnas.84.23.8740 3479811 PMC299622

[B64] Saito H. Tanaka K. Isono H. Yasuda M. Mikami A. (1989). Directionally selective response of cells in the middle temporal area (MT) of the macaque monkey to the movement of equiluminous opponent color stimuli. *Exp. Brain Res.* 75 1–14. 2707344 10.1007/BF00248524

[B65] Schmid A. M. (2008). The processing of feature discontinuities for different cue types in primary visual cortex. *Brain Res.* 1238 59–74. 10.1016/j.brainres.2008.08.029 18771659 PMC2602799

[B66] Seidemann E. Poirson A. B. Wandell B. A. Newsome W. T. (1999). Color signals in area MT of the macaque monkey. *Neuron* 24 911–917. 10.1016/s0896-6273(00)81038-7 10624954

[B67] Simoncelli E. P. Heeger D. J. (1998). A model of neuronal responses in visual area MT. *Vision Res.* 38 743–761. 10.1016/s0042-6989(97)00183-1 9604103

[B68] Soriano M. Spillmann L. Bach M. (1996). The abutting grating illusion. *Vision Res.* 36 109–116. 10.1016/0042-6989(95)00107-b 8746248

[B69] Spillmann L. Dresp B. (1995). Phenomena of illusory form: can we bridge the gap between levels of explanation? *Perception* 24 1333–1364. 10.1068/p241333 8643336

[B70] Stone L. S. Thompson P. (1992). Human speed perception is contrast dependent. *Vision Res.* 32 1535–1549. 10.1016/0042-6989(92)90209-2 1455726

[B71] Takeuchi T. De Valois K. K. Hardy J. L. (2003). The influence of color on the perception of luminance motion. *Vision Res.* 43 1159–1175. 10.1016/s0042-6989(03)00086-5 12705956

[B72] Thiele A. Dobkins K. R. Albright T. D. (1999). The contribution of color to motion processing in macaque middle temporal area. *J. Neurosci.* 19 6571–6587. 10.1523/jneurosci.19-15-06571.1999 10414985 PMC6782820

[B73] Thompson P. (1982). Perceived rate of movement depends on contrast. *Vision Res.* 22 377–380. 10.1016/0042-6989(82)90153-5 7090191

[B74] Tolias A. Keliris G. A. Smirnakis S. Logothetis N. K. (2005). Neurons in macaque area V4 acquire directional tuning after adaptation to motion stimuli. *Nat. Neurosci.* 8 591–593. 10.1038/nn1446 15834417

[B75] Troscianko T. Fahle M. (1988). Why do isoluminant stimuli appear slower. *J. Opt. Soc. Am. A* 5 871–880. 10.1364/josaa.5.000871 3404317

[B76] Von der Heydt R. Peterhans E. (1989). Mechanisms of contour perception in monkey visual cortex 1. Lines of pattern discontinuity. *J. Neurosci.* 9 1731–1748. 10.1523/jneurosci.09-05-01731.1989 2723747 PMC6569817

[B77] Von der Heydt R. Peterhans E. Baumgartner G. (1984). Illusory contours and cortical neuron responses. *Science* 224 1260–1262. 10.1126/science.6539501 6539501

[B78] Wandell B. A. Poirson A. B. Newsome W. T. Baseler H. A. Boynton G. M. Huk A. (1999). Color signals in human motion-selective cortex. *Neuron* 24 901–909. 10.1016/s0896-6273(00)81037-5 10624953

[B79] Webster M. A. (2015). Visual adaptation. *Annu. Rev. Vis. Sci.* 1 547–567. 26858985 10.1146/annurev-vision-082114-035509 PMC4742349

[B80] Willis A. Anderson S. J. (2002). Colour and luminance interactions in the visual perception of motion. *Proc. R. Soc. Lond. B Biol. Sci.* 269 1011–1016. 10.1098/rspb.2002.1985 12028757 PMC1690987

[B81] Yin J. Gong H. An X. Chen Z. Lu Y. Andolina I. M. (2015). Breaking cover: neural responses to slow and fast camouflage-breaking motion. *Proc. R. Soc. Lond. B Biol. Sci.* 282:20151182. 10.1098/rspb.2015.1182 26269500 PMC4632627

[B82] Yoshizawa T. Mullen K. T. Baker C. L. (2000). Absence of a chromatic linear motion mechanism in human vision. *Vision Res.* 40 1993–2010. 10.1016/s0042-6989(00)00069-9 10828467

[B83] Zeki S. M. (1978). Uniformity and diversity of structure and function in rhesus monkey prestriate visual cortex. *J. Physiol. Lond.* 277 273–290. 10.1113/jphysiol.1978.sp012272 418176 PMC1282389

