# Supplementary material for: The Serpentine Illusion: A Visual Motion Illusion Induced by Phase-Shifted Line Gratings
Source: Front Neurosci. 2020 Dec 7;14:612153. doi: 10.3389/fnins.2020.612153 (PMC7793819; doi:10.3389/fnins.2020.612153)
Supplement: Supplementary file 8 [file Data_Sheet_1.PDF]

frontiers

Supplementary Material

1 Supplementary Figures

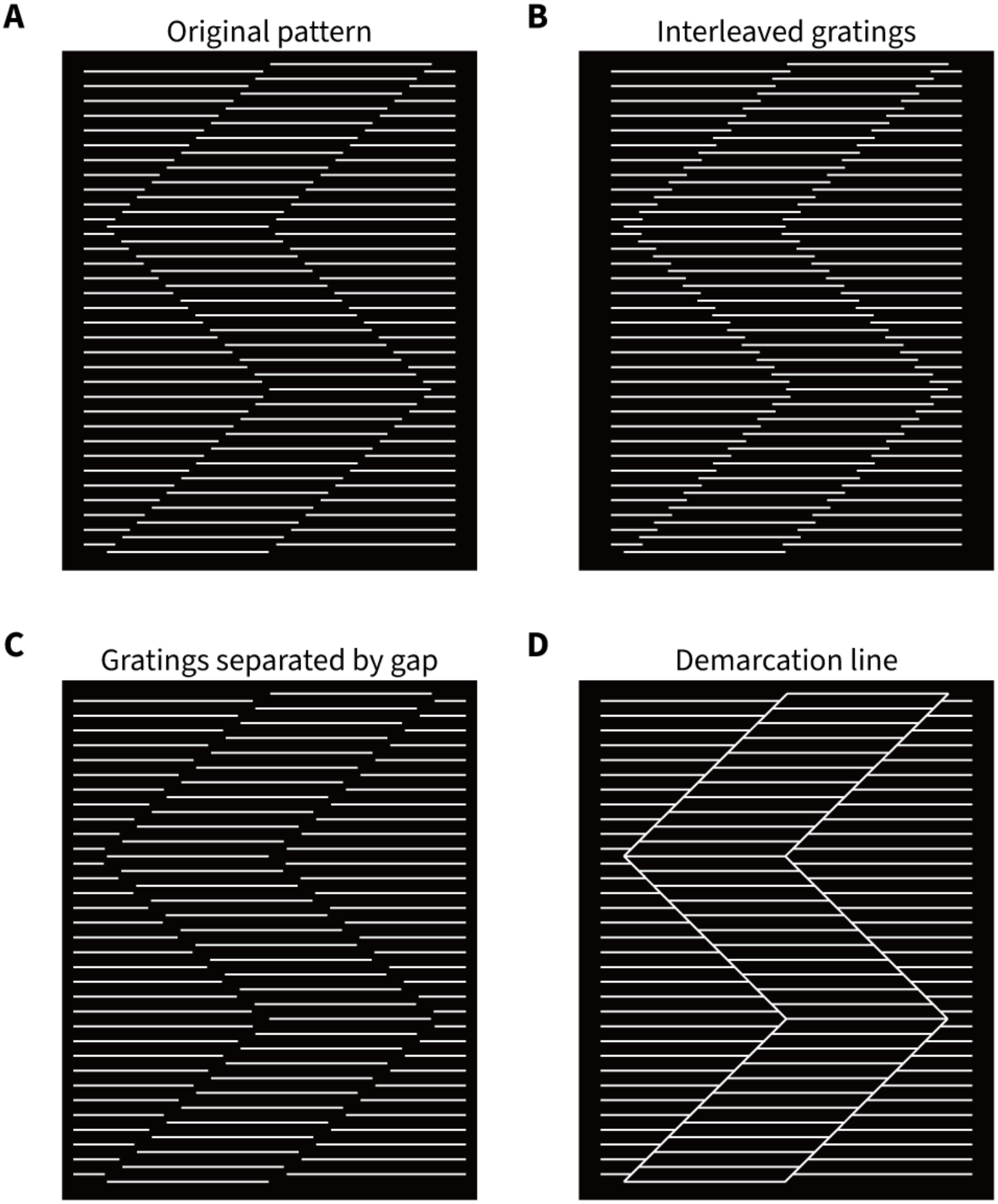

Supplementary figure 1. Modified stimulus patterns. (**A**) Original Serpentine Illusion pattern. (**B**) Grating lines are interleaved; no illusory motion perceived. (**C**) Gratings lines are separated by a gap; no illusory motion perceived. (**D**) Abutting line-ends are overlaid by a real contour; no illusory motion perceived.

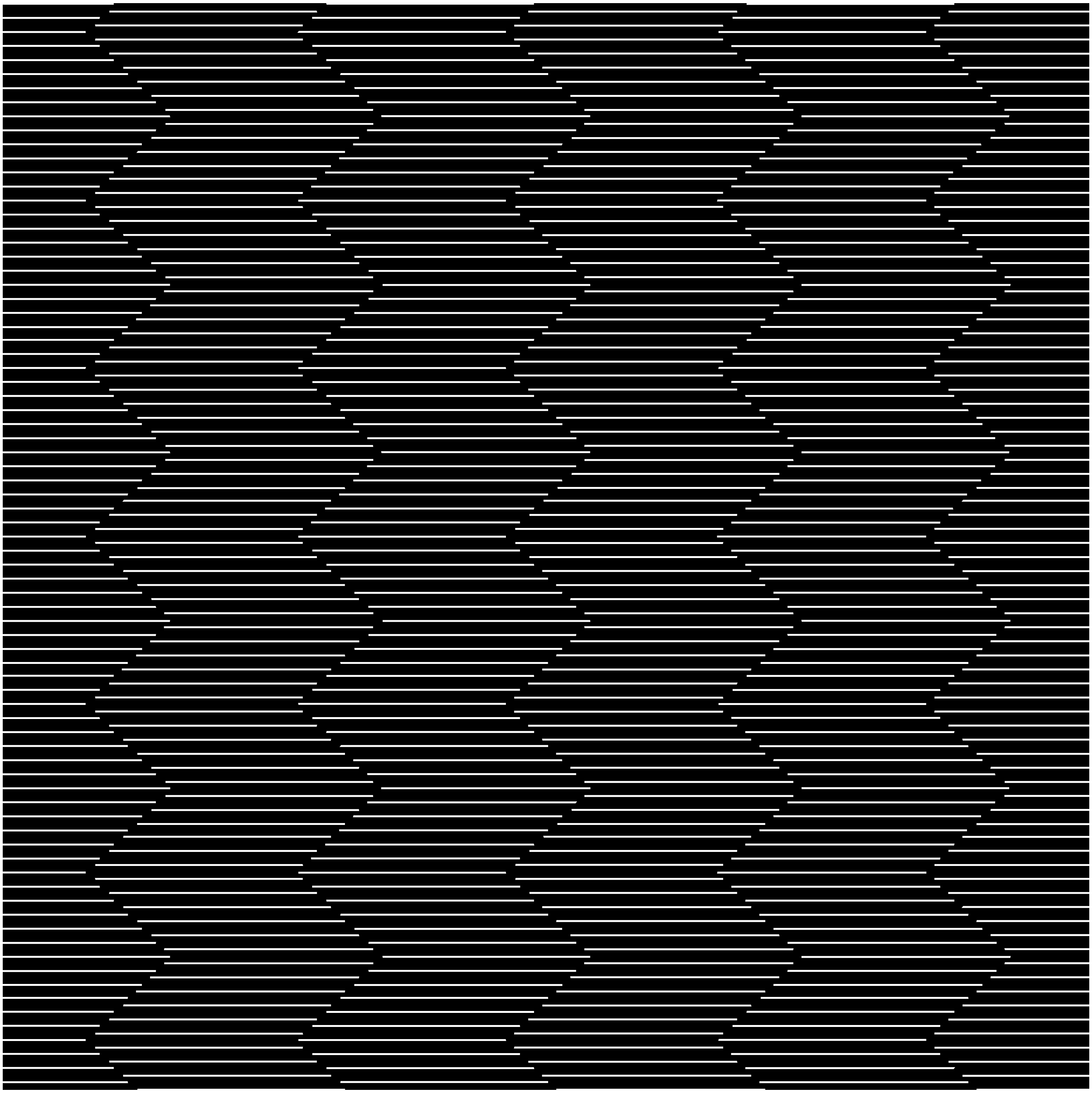

Supplementary figure 2. Stimulus pattern eliciting vivid diagonal illusory motion, using the parametric values taken from **Table 1**.

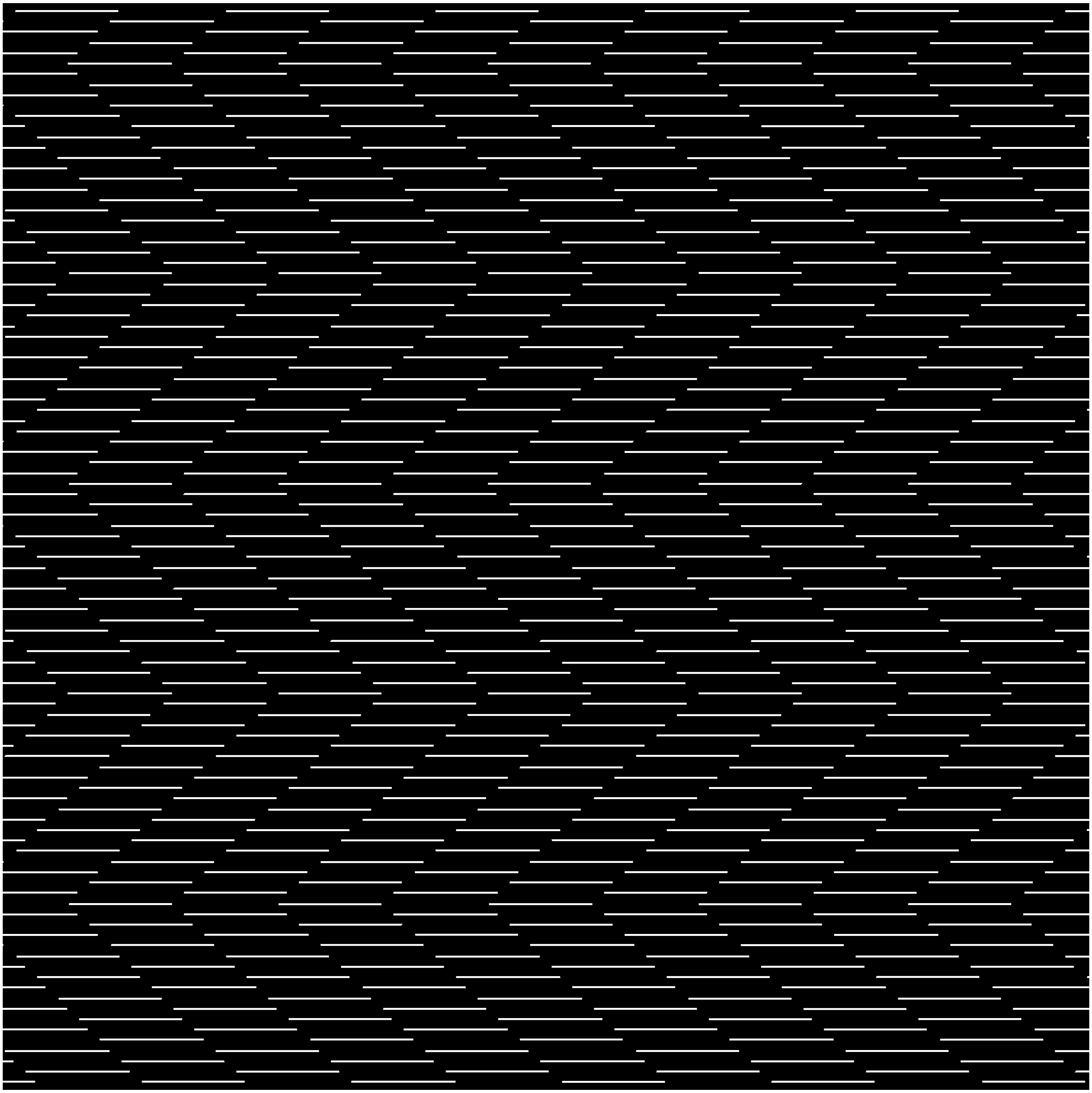

Supplementary figure 3. Stimulus pattern eliciting vivid diagonal and lateral motion, using the parametric values taken from **Table 1**.

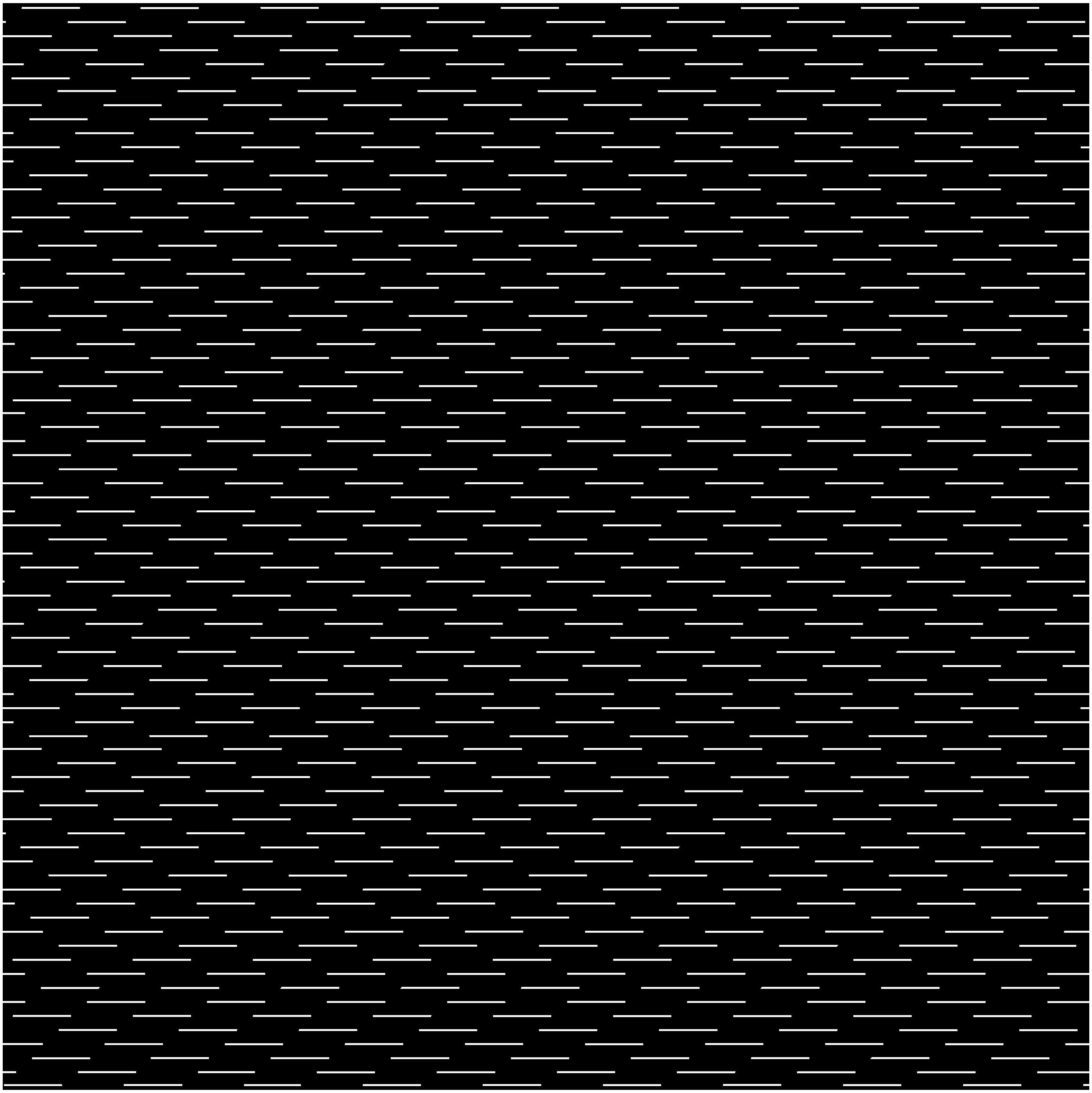

Supplementary figure 4. Stimulus pattern eliciting vivid lateral illusory motion, using parametric values taken from **Table 1**.

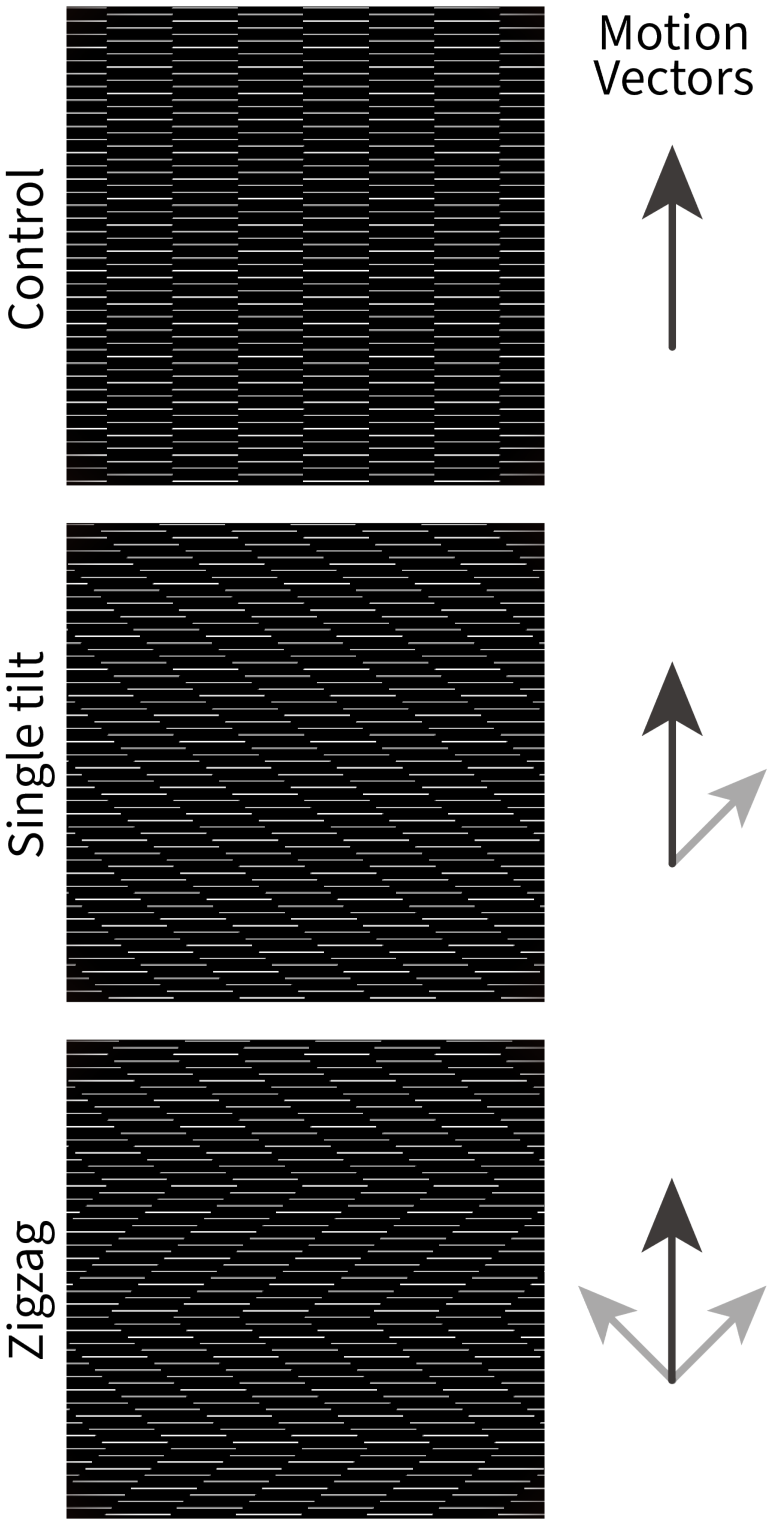

Supplementary figure 5. Serpentine illusory stimulus patterns that provide input to the spatio-temporal energy model. We tested patterns which optimized either diagonal, lateral or both-motions as estimated from human perception (**Table 1**; both-motions example is illustrated here). We made control (top), single-tilt (middle) and the full zigzag variants (bottom) which predict different motion vector (right) tuning curve transformations for model neurons.

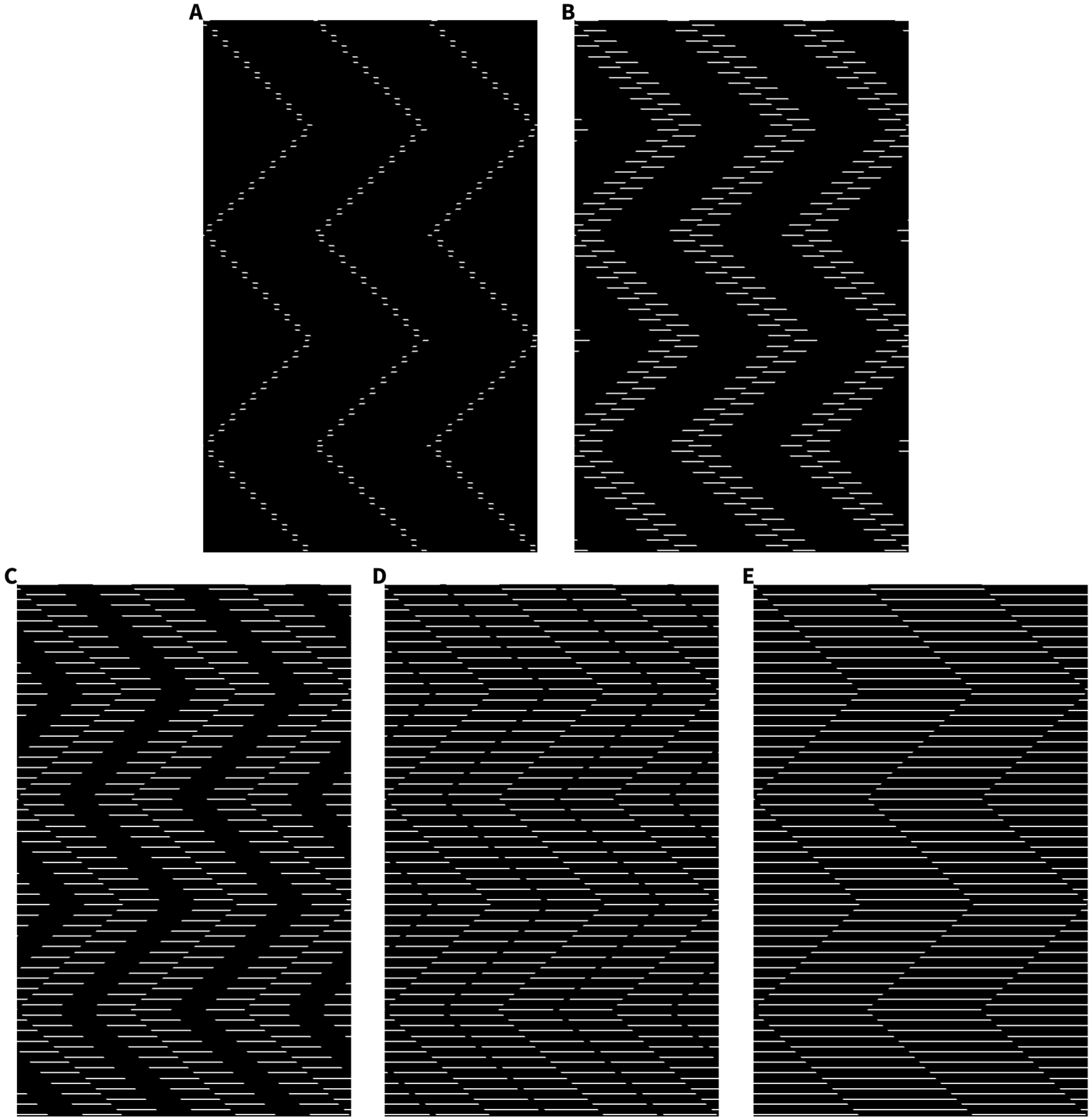

Supplementary figure 6. Modified stimulus patterns. (**A**) Modified pattern with end points only, grating lines were masked; no illusory motion perceived. (**B**) Modified pattern with ½ of the grating lines masked; very weak illusory motion perceived. (**C**) Modified pattern with ⅓ of the grating lines masked; weak illusory motion perceived. (**D**) Modified pattern with a mask of thin zigzag lines; illusory motion perceived, but weaker than that in (**E**). (**E**) Original Serpentine Illusion pattern.

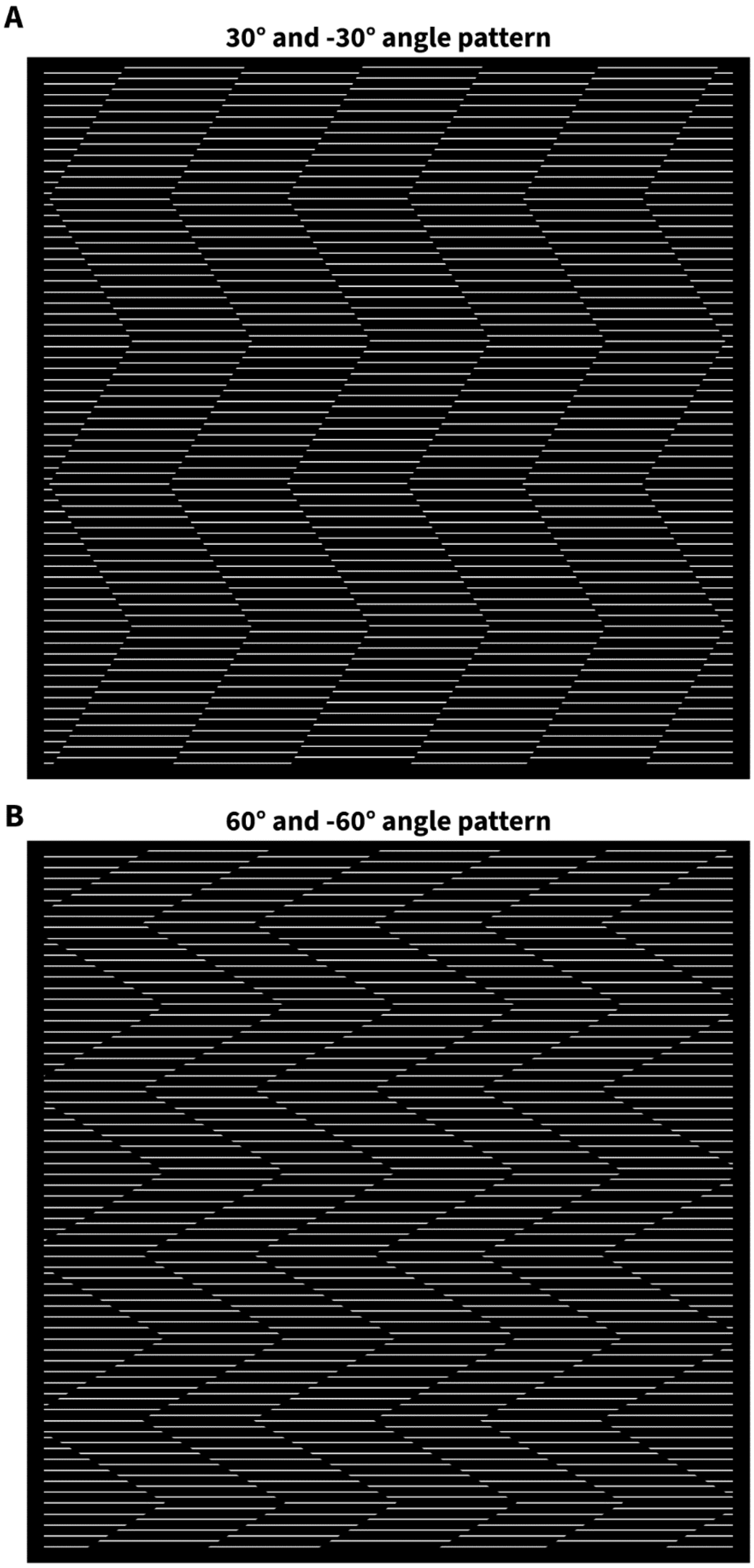

Supplementary figure 7. The orientation of the zigzagging illusory contours relative to the horizontal grating lines are changed from ± 45° in Figure 1 to (**A**) ± 30° and (**B**) ± 60°.

2 Supplementary video legends:

Supplementary video 1. The original Serpentine Illusion stimulus pattern. While keeping fixation at the center cross, physical upwards and downwards motion of the pattern elicits strong illusory diagonal and lateral motion perception across the pattern.

Supplementary video 2. Stimulus pattern that generates optimal diagonal motion.

Supplementary video 3. Stimulus pattern that generates optimal diagonal and lateral motions.

Supplementary video 4. Stimulus pattern that generates optimal lateral motion.

Supplementary video 5. Control stimulus patterns with overlaps between endpoints.

Supplementary video 6. Control stimulus patterns with gaps between endpoints.

Supplementary video 7. Control stimulus patterns with physical lines overlaid on endpoints.
